# Guard cell photorespiration controls stomata behavior and development

**DOI:** 10.1111/nph.71137

**Published:** 2026-03-26

**Authors:** Hu Sun, Inken Thiemann, Nils Schmidt, Johannes Kromdijk, Tracy Lawson, Martin Hagemann, Stefan Timm

**Affiliations:** ^1^ Plant Physiology Department University of Rostock Albert‐Einstein‐Straße 3 Rostock D‐18059 Germany; ^2^ Department of Plant Sciences University of Cambridge Downing Street Cambridge CB23EA UK; ^3^ University of Essex Wivenhoe Park Colchester CO4 3SQ UK; ^4^ Department of Plant Biology Institute for Genomic Biology, University of Illinois at Urbana Champaign 1206 W Gregory Urbana IL 61801 USA

**Keywords:** 2‐phosphoglycolate phosphatase, Arabidopsis, environmental acclimation, photosynthesis, plant growth

## Abstract

- Photorespiration is traditionally viewed as a limitation to photosynthetic efficiency. However, it is mandatory for safeguarding the Calvin–Benson–Bassham cycle from inhibitory byproducts through Rubisco‐mediated oxidative misfire and is tightly integrated with primary metabolism. Whether photorespiratory metabolism directly regulates guard cell function and stomatal behavior remains a matter of intense debate.
- We manipulated the photorespiration‐restricted pathway entry enzyme 2‐phosphoglycolate phosphatase (PGLP1) specifically in *Arabidopsis thaliana* (Arabidopsis) guard cells and assessed effects on growth, photosynthesis, carbohydrate allocation, cell‐specific H_2_O_2_ accumulation, and stomatal traits under photorespiratory conditions, including exogenous 2‐phosphoglycolate (2‐PG) feeding.
- Altered guard cell PGLP1 protein expression consistently affected plant growth, photosynthetic performance, and stomatal movement. PGLP1 perturbation induced guard cell‐specific starch and H_2_O_2_ accumulation patterns, both of which are central components involved in driving optimal stomatal behavior. Further, altered stomatal size was observed, a phenotype that was recapitulated by external 2‐PG application to wild‐type plants.
- Efficient photorespiratory metabolism is essential for proper guard cell function and acclimation to changing CO_2_ : O_2_ ratios. Our findings uncover a direct metabolic link between photorespiration and stomatal behavior, revealing an unexpected role for this ancient pathway in controlling gas exchange, photosynthesis, and potentially plant productivity and resilience.

## Introduction

Plant photosynthesis converts atmospheric CO_2_ into sugars that sustain the global food chain. Under the present‐day high O_2_ : CO_2_ ratio, photosynthetic efficiency is limited by Rubisco's oxygenase activity, which generates 2‐phosphoglycolate (2‐PG), a potent inhibitor of the Calvin–Benson–Bassham (CBB) cycle enzymes sedoheptulose‐1,7‐bisphosphatase (SBPase) and triosephosphate isomerase (TPI) (Flügel *et al*., 2017; Li *et al*., 2019). Photorespiration exclusively removes 2‐PG to prevent metabolic inhibition, yet it is energetically costly and releases previously fixed CO_2_ and NH_3_^+^ (Walker *et al*., 2016). Beyond its essential repair function, photorespiration supports *de novo* nitrogen and sulfur assimilation, amino acid biosynthesis, one‐carbon metabolism, and redox balance, thereby contributing to acclimation under fluctuating conditions (Foyer *et al*., 2009; Busch, 2020; Timm *et al*., 2025b). These Janus‐faced roles, protective but metabolically expensive, make photorespiration a key target for improving plant productivity under current and future climates (Cavanagh *et al*., 2022; Smith *et al*., 2025).

Leaf‐mesophyll photorespiration is well‐characterized, but its function in specific cell types remains largely unexplored. Although the pathway is highly compartmentalized, spanning chloroplasts, mitochondria, peroxisomes, cytosol, and vacuoles (Lin & Tsay, 2023; Jiang *et al*., 2025; Timm *et al*., 2025b), its interaction with other metabolic routes is not well resolved. Recent isotopically non‐stationary metabolic flux analyses (INST‐MFA) showed that a substantial fraction of carbon exits the canonical cycle, feeding other biosynthetic routes including C1‐carbon metabolism, mainly as serine and glycine (Fu *et al*., 2023; Fu & Walker, 2024; Gashu *et al*., 2025). This implies that certain reactions exert disproportionate control over the photorespiratory flux and may regulate carbon utilization and partitioning in distinct compartments. Genetic studies have highlighted two key control points: mitochondrial glycine decarboxylase (GDC) and chloroplast‐localized 2‐PG phosphatase 1 (PGLP1). Both enzymes share a strong positive correlation with the photorespiratory flux, CBB cycle operation, starch biosynthesis, and plant growth, making them attractive targets for improved yield (Timm *et al*., 2012a, 2015, 2025a; Flügel *et al*., 2017).

Photorespiratory rates are determined by the CO_2_ : O_2_ ratios in the chloroplasts, which largely depend on opening of stomata, formed by pairs of guard cells (GC) in the leaf epidermis. In addition to flux of CO_2_ and O_2_, stomatal opening regulates water vapor movements, balancing carbon gain with water conservation (Vavasseur & Raghavendra, 2005; Santelia & Lawson, 2016; Pankasem *et al*., 2024). Stomatal size and density are inversely related and determine maximum stomatal conductance (*g*_smax_); small, numerous stomata support fast stomatal kinetics (Franks *et al*., 2009) and higher gas exchange than few, large stomata. Across evolutionary timescales, high atmospheric CO_2_ concentrations ([CO_2_]) favored fewer, larger stomata, whereas low [CO_2_] selected for smaller, denser stomata (Franks *et al*., 2009; Drake *et al*., 2013; Inoue & Kinoshita, 2017). On daily timescales, stomatal size and density will not change, but GC respond dynamically to environmental cues, including light, internal [CO_2_], temperature, humidity, and water availability. Light is a dominant driver, red light triggers GC osmoregulation and transmits mesophyll signals that align stomatal opening with photosynthetic demand at higher light intensities (Jezek & Blatt, 2017). It has been shown that red light‐induced stomatal opening involves sucrose‐dependent upregulation of H^+^‐ATPase phosphorylation, to increase its activity specifically in guard cells, and to drive stomatal opening (Ando & Kinoshita, 2018; Zait *et al*., 2025). Blue light acts independently of photosynthesis, directly via phototropin kinases to induce rapid opening at low fluence rates, particularly at dawn and during transient sunlight fluctuations, maximizing carbon assimilation (Kinoshita *et al*., 2001; Lawson & Matthews, 2020; Taylor *et al*., 2024). Stomatal closure is mainly achieved via the plant hormone abscisic acid (ABA), maintaining overall plant water status (Vavasseur & Raghavendra, 2005; Santelia & Lawson, 2016).

Intercellular CO_2_ (*C*_i_) is regarded as another important factor controlling stomata. For example, *C*_i_ elevation with decreasing photosynthesis, darkness or via raised external [CO_2_] promotes stomatal closure, whereas light‐dependent draw‐down of *C*_i_ via photosynthetic CO_2_ fixation maintains opening (Lawson *et al*., 2003). Further, a recent report also suggests warming as a factor involved in lowering *C*_i_, and in turn triggering stomatal opening, via stimulation of photosynthesis (Pankasem *et al*., 2024). Rising global [CO_2_] reduces stomatal aperture and density, lowering conductance and conserving water, yet potentially increasing leaf temperature under drought. At the molecular level, CO_2_ responses require the protein kinase high leaf temperature 1 (HT1) (Hashimoto *et al*., 2006; Hashimoto‐Sugimoto *et al*., 2016) and converge with ABA signaling, because elevated [CO_2_] increases guard cell ABA to induce closure. Thus, stomatal behavior integrates red/blue light signals, CO_2_ feedback, and hormonal cues to balance carbon gain, water use, and thermal stability (Lefebvre *et al*., 2005; Ding *et al*., 2016; Engineer *et al*., 2016).

In addition to photorespiration, the activity of Rubisco carboxylation and the flux through the CBB cycle also respond to fluctuating CO_2_ : O_2_ ratios. It has been shown that SBPase activity might be a control point of the CBB cycle flux, because its overexpression in plants enhanced photosynthesis and yield (Driever *et al*., 2017). Comparable effects have been observed in overexpressors of key photorespiratory enzymes such as GDC and PGLP1 (Timm *et al*., 2012a, 2015, 2025a; Flügel *et al*., 2017), whereas impairment of photorespiration diminished productivity (Timm *et al*., 2012b; Betti *et al*., 2016). Interestingly, the recently reported GC‐specific manipulation of GDC suggested a functional link between mitochondrial photorespiratory metabolism and stomatal regulation (Sun *et al*., 2025). If this finding is specific to mitochondria or GDC, releasing CO_2_ during photorespiration, thereby eventually affecting *C*_i_, remains unknown. However, initial pharmacological studies on photorespiratory enzymes in epidermal peels indirectly supported a functional interaction between the activity of certain photorespiratory enzymes and stomatal movements (Mortezazadeh *et al*., 2025). Surprisingly, the role of PGLP1, the photorespiration‐specific 2‐PG‐degrading enzyme, in GC is still unclear. Gaining insights into its physiological significance is interesting because of the reduced GC chloroplast count and size, alongside hints for the sink‐tissue‐like characteristics of GC, and the ongoing debate on the extent to which GC rely on their own internal photosynthesis (Lawson *et al*., 2014; Santelia & Lawson, 2016).

To address this question, we analyzed the impact of GC‐specific manipulation of the central photorespiratory enzyme, chloroplastidal PGLP1. The transgenic lines with increased and decreased GC‐specific *PGLP1* expression were assessed to study its role in GC metabolism and impact on stomata function, photosynthesis and biomass accumulation. By elucidating the role of photorespiration in these specialized cells, our work not only provides new insights into its significance for GC metabolism but also provides the foundation for new strategies to engineer crops with enhanced growth, water‐use efficiency, and yield, key traits to meet the challenges of plant production under increasing climate change.

## Materials and Methods

### Plant growth conditions and biomass quantification

*Arabidopsis thaliana* (L.) Heynh., ecotype Columbia.0 (Col.0), was used as the wild‐type control and as the background for generating GC‐specific overexpression and antisense repression lines of photorespiratory 2‐phosphoglycolate (2‐PG) phosphatase 1 (PGLP1; At5g36700) and the CBB cycle enzyme sedoheptulose‐1,7‐bisphosphatase (SBPase; At3g55800). Seeds were surface sterilized using chlorine gas (generated by mixing 25 ml of 12% sodium hypochlorite with 1.5 ml concentrated HCl in a sealed desiccator) for 3 h. Sterilized seeds were sown on a soil–vermiculite mixture (4 : 1, v/v; MiniTray soil, Einheitserdewerk, Uetersen, Germany), stratified at 4°C for 48 h in darkness to break dormancy, and then transferred to growth chambers. Plants were cultivated under controlled environmental conditions (Percival or SANYO growth chambers) with the following standard settings, unless otherwise stated: photoperiod; 12 h : 12 h, light : dark, temperature; 22°C (day) : 20°C (night), light intensity; 120–140 μmol m^−2^ s^−1^ (cool‐white fluorescent lamps), relative humidity; *c*. 70%, CO_2_ concentration; 400 ppm (air) or for high CO_2_ (HC) treatments; 3000 ppm, with otherwise identical conditions. Plants were watered to maintain uniform soil moisture and fertilized weekly with 0.2% Wuxal liquid fertilizer (Aglukon, Düsseldorf, Germany). Pots were randomized within the chamber weekly to minimize positional effects. Unless otherwise specified, all physiological experiments were performed using plants at growth stage 5.1 (Boyes *et al*., 2001).

Selected quantitative growth parameters were determined from all side‐by‐side grown genotypes, using 10 independent biological replicates per genotype. Rosette diameters were measured as the maximum distance across the fully expanded rosette and only fully expanded leaves were considered to determine the leaf count. Next, rosettes were excised, weighed immediately to determine fresh weight, dried at 100°C to constant weight (*c*. 24–30 h), and reweighed for dry biomass determination. For 2‐PG feeding assays, wild‐type plants were grown *in vitro* on freshly prepared half‐strength Murashige and Skoog (MS) medium (pH 5.7), supplemented with 0, 10, 50, or 100 μM of 2‐PG (Sigma‐Aldrich, Taufkirchen, Germany). Leaves of seedlings at growth stage 1.04 (Boyes *et al*., 2001) from at least three independent plates per treatment were used for microscopic analysis of stomatal parameters.

### Cloning and plant transformation procedures

Guard cell‐specific transgenic Arabidopsis lines were generated to achieve overexpression or antisense‐mediated reduction of *PGLP1* and *SBPase* expression. The binary plant transformation vector pG0229:AtGC1:35STer, containing the guard cell‐specific GC1 promoter (Yang *et al*., 2008; Sun *et al*., 2025), served as the expression backbone. The full coding sequence (CDS) of *Solanum lycopersicum PGLP1* (*SlPGLP1*; 1119 bp) was synthesized *de novo* (BaseClear, Leiden, the Netherlands). The CDS of Arabidopsis SBPase (*AtSBPase*; 1182 bp) was PCR‐amplified from Col.0 cDNA using primers P967 and P968 (sequences listed in Supporting Information Table S9) with a proof‐reading DNA polymerase and cloned into pJET2.1 (ThermoFisher Scientific, Schwerte, Germany) for sequence verification and amplification. The coding fragments were excised from their entry vectors using *Bam*HI (*SlPGLP1*) and *Xma*I (*AtSBPase*), respectively, and ligated into pG0229:AtGC1:35STer in sense and antisense orientations to create overexpression constructs pG0229:AtGC1:SlPGLP1_sense:35STer and pG0229:AtGC1:AtSBPase_sense:35STer and antisense constructs pG0229:AtGC1:SlPGLP1_antisense:35STer and pG0229:AtGC1:AtSBPase_antisense:35STer (see Supporting Information Figs S1, S3). All final constructs were verified by sequencing (Microsynth, Göttingen, Germany). Subsequently, the constructs were introduced into *Agrobacterium tumefaciens* GV3101 + pSOUP, the drug‐resistant colonies verified via standard PCR procedures, and used for Arabidopsis floral dip transformation (Clough & Bent, 1998). T1 seeds were surface sterilized and selected on half‐strength MS media supplemented with 20 μg ml^−1^ phosphinothricin (BASTA). Resistant seedlings were transplanted to soil, PCR‐verified for the presence of the transgene, and propagated to homozygous T3 or T4 lines, used for all physiological experiments. For comprehensive characterization, two independent *SlPGLP1* and three independent *AtSBPase* overexpression and antisense lines were used.

### Verification of transgenic lines and immunological studies

Genomic DNA was isolated from rosette leaves according to standard procedures. Transgene integration was verified by PCR using primers specific for the exogenous *SlPGLP1* (P953 for sense and P954 for antisense orientation) or *AtSBPase* (P967 for sense and P968 for antisense orientation) in combination with the *AtGC1* promoter primer P950. PCR reactions were performed using a standard DNA polymerase under the following conditions: 94°C for 1 min, 58°C for 1 min, 72°C for 2 min, for 35 cycles. DNA integrity was confirmed by amplification of the *S16* gene (At2g09990) using primers P444 and P445 under identical cycling conditions, except for a 30 s extension step (see Figs S1C and S3C).

Transcript accumulation of *SlPGLP1* and *AtPGLP1* was assessed by semiquantitative RT‐PCR. Total RNA (2.5 μg) was extracted using the Nucleospin RNA Plant Kit (Macherey‐Nagel, Düren, Germany) and treated with DNaseI to remove genomic DNA contamination. First‐strand cDNA synthesis was performed with the RevertAid cDNA Synthesis Kit (Thermo Fisher Scientific, Osterode, Germany) using oligo(dT) primers. Diagnostic transcript fragments were amplified using primers P974/P975 (*SlPGLP1*, 336 bp) and P977/P978 (*AtPGLP1*, 288 bp). Amplification of *S16* (432 bp) with primers P444/P445 served as an internal control. PGLP1 and SBPase protein abundance was determined by immunoblotting. Total soluble protein was extracted from mesophyll and guard cell‐enriched fractions from the same leaves, and equal amounts (5 μg per lane) were separated by SDS‐PAGE and electroblotted onto polyvinylidene difluoride (PVDF) membranes. Blots were probed with specific anti‐PGLP1 (Flügel *et al*., 2017) or anti‐SBPase (Dunford *et al*., 1998) antibodies. GDC‐H and RbcL antibodies (Agrisera, Vännäs, Sweden) were used as loading and normalization controls. Signal detection was performed via chemiluminescence, and densitometric quantification was carried out using imagej (https://imagej.net/) from at least three independent replicates.

### Isolation of mesophyll and guard cell protein extracts

Mesophyll‐ and guard cell‐enriched fractions were obtained as described previously (Lawrence *et al*., 2018) with minor modifications. Fully expanded leaves from 5‐ to 6‐wk‐old plants grown under standard conditions were harvested at mid of the day (*c*. 6 h illumination). Transparent adhesive tape was applied to either the abaxial (for guard cell enrichment) or adaxial (for mesophyll enrichment) leaf surface. Peels (*c*. 20–50 per genotype, as a mixture from at least four biological replicates) were gently removed, pooled by fraction, and immediately frozen in liquid nitrogen. Protein extraction was performed essentially as described earlier (Lawrence *et al*., 2018) and protein concentrations were determined using the BCA Protein Assay Kit (Thermo Scientific, Osterode, Germany) according to the manufacturer's instructions, with bovine serum albumin as standard.

### Guard cell properties and guard cell starch content

To determine diagnostic parameters associated with GC morphology, epidermal peels were prepared from fully expanded rosette leaves of plants grown under standard conditions, harvested at midday (*c*. 6 h of illumination). Transparent nail polish was applied to the abaxial surface of each leaf and allowed to dry for 10 min. The epidermis was gently peeled off, mounted in water on microscope slides, and covered with a coverslip. Four biological individuals per genotype were analyzed. GC parameters (area, length, width, density and index) were measured using an Olympus U‐LH100HG microscope (Olympus Corporation, Tokyo, Japan) and the manufacturer's image analysis software.

Starch content in GC was assessed in epidermal peels harvested at midday (*c*. 6 h of illumination) following propidium iodide staining as described before (Flütsch *et al*., 2018). Four biological replicates per genotype were used, with 10 guard cells randomly selected per stained peel. Fluorescence images were acquired using a Keyence BZ‐X800 fluorescence microscope (Keyence Deutschland GmbH, Neu‐Isenburg, Germany) equipped with a Plan Fluorite 20‐100× LD PH objective at 100× magnification. Fluorescence was visualized with the BZ‐X GFP filter cube (exposure time: 1/70 s) and captured with bz‐x800 viewer software. Quantitative analysis of GC starch was performed by measuring fluorescence intensity per cell using the manufacturer's software.

### Guard cell H_2_O_2_ content determination

Reactive oxygen species (ROS), primarily H_2_O_2_, in GC were visualized using 2′,7′‐Dichlorodihydrofluorescein diacetate (H_2_DCFDA) fluorescence staining as described previously (Shi *et al*., 2024), using plants grown in air to stage 5.1 (Boyes *et al*., 2001). The lower epidermis was carefully excised and incubated in 100 μM H_2_DCFDA prepared in 10 mM Tris–HCl buffer (pH 7.2) in the dark for 10 min. Excess dye was removed, and the peels were washed three times with 10 mM Tris–HCl (pH 7.2). Fluorescence images were captured using a Keyence BZ‐X800 fluorescence microscope (Keyence Deutschland GmbH, Neu‐Isenburg, Germany) equipped with a Plan Fluorite 100× LD PH objective. H_2_DCFDA fluorescence was visualized using the GFP filter cube, and images were acquired with the bz‐x800 viewer software. Quantification of fluorescence intensity in GC was performed using the same software.

### Gas exchange measurements

Gas exchange was measured using LI‐6400 and LI‐6400XT Portable Photosynthesis Systems equipped with a 2 cm^−2^ LED leaf chamber fluorometer and red/blue light source (LI‐COR Biosciences, Lincoln, NE, USA). Before each measurement day, CO_2_ and H_2_O analyzers were calibrated according to the manufacturer's instructions. Fully expanded rosette leaves from plants grown under standard conditions (light intensity *c*. 120–140 mmol m^−2^ s^−1^) were clamped in the cuvette and pre‐acclimated for 10 min at 1000 μmol m^−2^ s^−1^ photosynthetic photon flux density (PPFD; 10% blue light) to reach stable steady‐state photosynthesis. Basic settings were as follows: 25°C block temperature, 400 μmol mol^−1^ CO_2_, 300 μmol s^−1^ flow rate, and *c*. 50–70% relative humidity. CO_2_ response (*A*/*C*_i_) curves were measured under constant 21% O_2_ and varying CO_2_ concentrations as follows: 400, 300, 200, 100, 50, 25, 0, and 400 ppm. To determine the oxygen‐dependence of the net CO_2_ compensation point, the O_2_ concentration was adjusted to 3, 21, and 40% O_2_ (balanced with N_2_), using the gas mixing device GMS600 (QCAL Messtechnik, München, Germany). The net CO_2_ assimilation rate (*A*_N_), stomatal conductance (*g*_s_), intercellular CO_2_ concentration (*C*_i_), transpiration rate (*E*), intrinsic water‐use efficiency (WUE_int_), and CO_2_ compensation point (*Γ*) were calculated by the LI‐6400 and excel software. O_2_ inhibition of *A*_N_ was calculated from measurements at 21 and 40% O_2_ using the equation: O_2_ inhibition = (*A*_21_–*A*_40_)/*A*_21_ × 100. Calculation of *γ* (measure of the photorespiratory CO_2_‐release) was performed by linear regression of the *Γ*‐vs‐O_2_ concentration curves and is given as slopes of the respective functions. Light response curves were measured under ambient CO_2_ and O_2_ levels (10 min acclimation at 1000 μmol m^−2^ s^−1^ PPFD), followed by stepwise reduction of PPFD to 1600, 1200, 800, 400, 200, 100, 50, 25, and 0 μmol m^−2^ s^−1^, allowing 2–3 min for stabilization at each step. At least six independent plants per genotype were measured and values are given as means ± SD.

### Chlorophyll fluorescence measurements

Selected PSI and PSII parameters associated with photosynthetic light reactions were determined by standard Chl fluorescence measurements on a Dual‐PAM‐100 (Heinz Walz, Effeltrich, Germany). Chlorophyll fluorescence measurements were performed on the adaxial leaf surface. PSI activity was determined by monitoring P700 absorbance, which reflects excitation across the entire leaf tissue, whereas PSII activity was assessed via Chl fluorescence, which predominantly originates from a defined layer of chloroplasts within the leaf mesophyll. Following 10 min dark adaptation, *F*_v_ : *F*_m_ (maximum quantum efficiency of PSII) and *P*_m_ (maximum photo‐oxidizable P700) values were recorded. Next, plants were exposed to 1000 μmol photons m^−2^ s^−1^ for 10 min to fully induce photosynthesis and, subsequently, light response curves were measured (PPFD: 1759, 1144, 757, 488, 236, 143, 62, 36, and 0 μmol photons m^−2^ s^−1^) at 400 ppm CO_2_ and 21% O_2_.

### Metabolite analysis

For absolute quantification of metabolites associated with primary metabolism, we used liquid chromatography coupled to tandem mass spectrometry (LC‐MS/MS) and gas chromatography analysis. Fully expanded rosettes were harvested under growth light at the end of the photoperiod (after 11 h illumination). All samples were collected within a 10‐min window to minimize variation, immediately quenched in liquid nitrogen, and stored at −80°C. Before further processing, the frozen material was lyophilized, and *c*. 2–3 mg dry weight per sample was aliquoted for extraction. For metabolite extraction and LC‐MS/MS measurements, we used LC‐MS grade chemicals and the procedure described before (Reinholdt *et al*., 2019). Measurements were carried out on a high‐performance liquid chromatograph mass spectrometer LC‐MS‐8050 system (Shimadzu, Kyoto, Japan) and the incorporated LC‐MS/MS method package for primary metabolites (v.2; Shimadzu). Selected soluble sugars and starch were measured on the gas chromatograph 6890 N GC System (Agilent Technologies, Waldbronn, Baden‐Württemberg, Germany) and spectrophotometrically, essentially as described previously (Sun *et al*., 2025). For each metabolite, absolute concentrations were determined using calibration curves generated from authentic standards measured in parallel. Results were normalized to dry weight and reported as nmol mg^−1^ DW (LC‐MS/MS) or μg g^−1^ DW (GC).

### Statistical analysis

We used the programs Microsoft excel and sigmaplot vol. 13.0 for data processing and graph generation, and CorelDraw (Graphics Suite, 2017; www.corel.com) was used for image compilation. Statistical differences were determined through analysis of variance (ANOVA; SPSS Statistics 27, IBM) using Tukey's Honestly Significant Difference (HSD) and Least Significant Difference (LSD) *post hoc* tests to determine significantly differing means between groups. The term significant is used here only if the change in question has been confirmed to be significant at the level of *P* < 0.05.

## Results

### Guard cell PGLP1 expression exerts control over growth and biomass accumulation

**Fig. 1:**
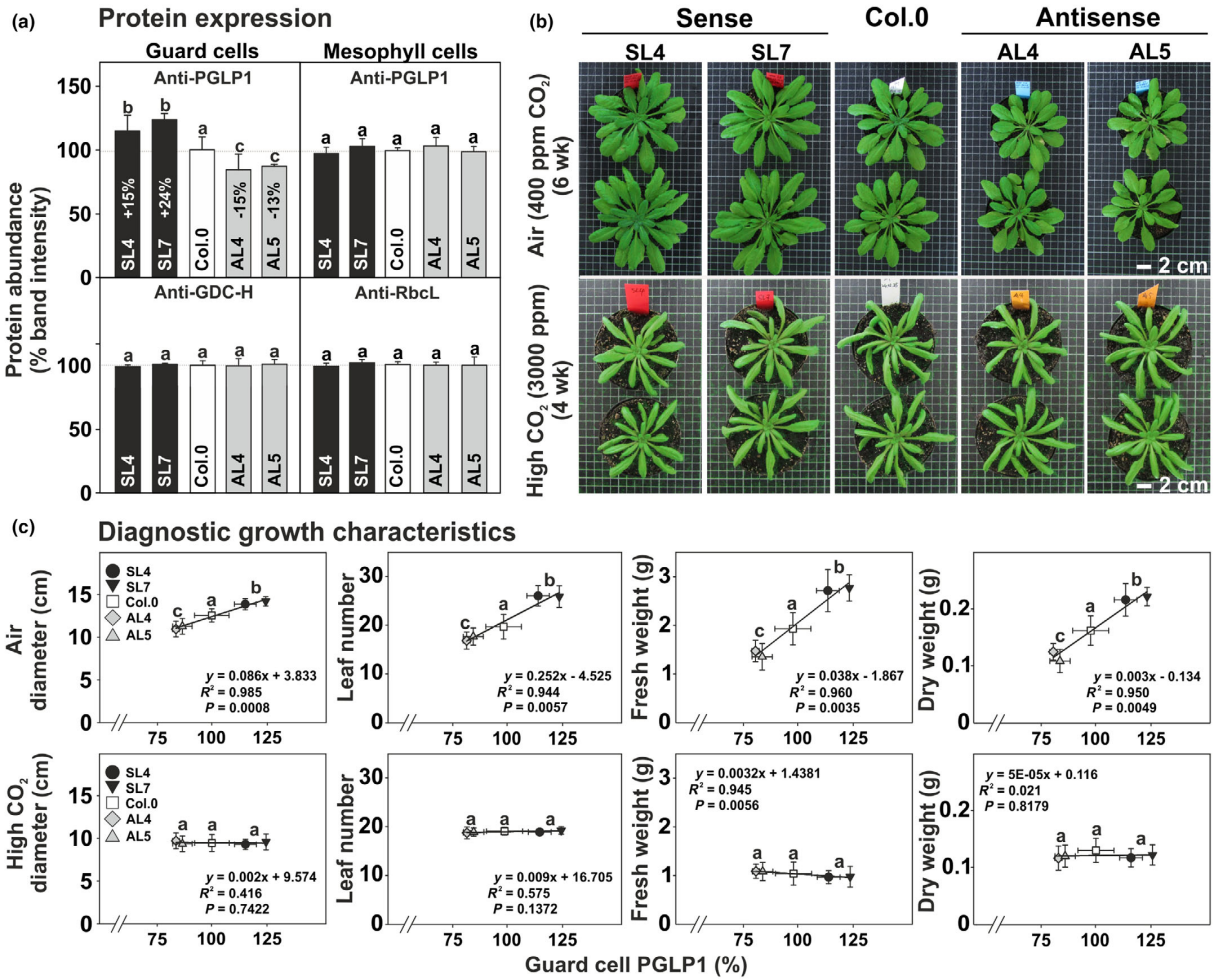
Protein expression and growth of Arabidopsis lines with GC‐specific *PGLP1* overexpression or antisense repression under ambient and elevated CO_2_ levels. (a) PGLP1 protein expression in GC (left panel) and MC (right panel). GDC‐H and RbcL protein amounts were quantified from the same membrane as control. (b) Photographs of the transgenic lines and the wild‐type after 6 wk in air (upper panel) or 4 wk in high CO_2_ (lower panel) with a 12 h : 12 h, day : night cycle. (c) Correlation plots of growth parameters and GC PGLP1 protein abundances in air (upper panel) and high CO_2_ (lower panel). Given are means ± SD of (a) three independent immunoblots and (c) six biological replicates (for full numerical growth data see Supporting Information Table S1). Values that do not share the same letter are significantly different from each other as determined by ANOVA. PGLP1, 2‐phosphoglycolate phosphatase 1; SL, sense lines; AL, antisense lines.

To determine the role of photorespiration, particularly 2‐PG degradation, in GC of the C_3_ plant Arabidopsis, we used the guard cell‐specific GC1 promoter (Yang *et al*., 2008) to specifically manipulate GC *PGLP1* expression (Fig. S1). Overexpression (sense lines: SL4 + 15% and SL7 + 24%) and antisense repression (antisense lines: AL4–15% and AL5–13%) of *PGLP1* were observed in GC, while PGLP1 protein abundances remained unaltered in mesophyll cells (MC) of the same leaves (Fig. 1a). Increased expression of *PGLP1* in GC stimulated, while antisense repression reduced the apparent growth of Arabidopsis under photorespiratory conditions (Fig. 1b). Diagnostic growth parameters followed this consistent pattern, as leaf number, rosette diameter, fresh and dry weights positively correlated with GC PGLP1 protein expression (Fig. 1c; Table S1). However, growth alterations relied on active photorespiration, as no growth differences were observed with plants grown in high CO_2_ (3000 ppm), strongly suppressing 2‐PG formation and photorespiration (Fig. 1b,c; Table S1).

### GC PGLP1 shapes photosynthetic CO_2_ assimilation and stomatal conductance

**Fig. 2:**
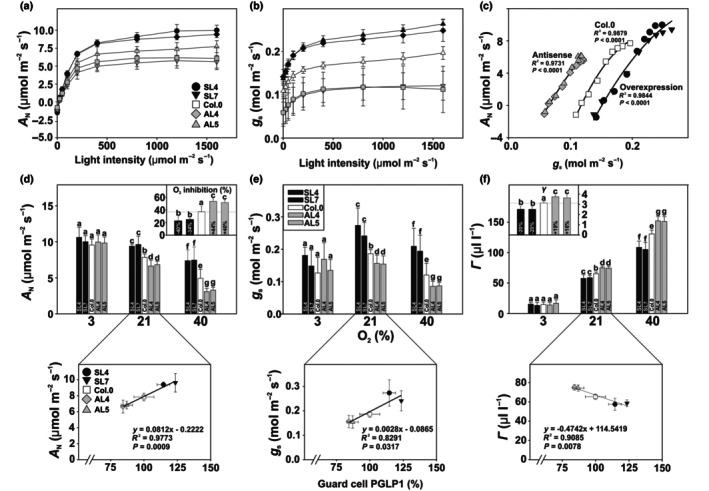
Photosynthetic gas exchange in Arabidopsis lines with GC‐specific *PGLP1* overexpression or antisense repression under different light and O_2_ levels. Plant was grown in air to stage 5.1 (Boyes *et al*., 2001) to determine photosynthetic gas exchange as functions of light intensity and O_2_ concentrations. Selected parameters of the light response curves are given as follows: (a) Net CO_2_ uptake rates (*A*_N_), (b) stomatal conductance (*g*_s_), and (c) correlation plot of *A*_N_ vs *g*_s_. Selected parameters of the CO_2_ response curves are presented as follows: (d) *A*_N_, including the oxygen inhibition of photosynthesis as inlet, (e) *g*_s_, and (f) CO_2_ compensation points (*Γ*), including the slope of the *Γ*‐vs‐O_2_ concentration functions (*γ*) as inlet. Correlation plots of *A*_N_, *g*_s_, and *Γ* at 21% O_2_ (air) are displayed at the bottom of each figure. Given are means ± SD of six biological replicates. Values that do not share the same letter are significantly different from each other as determined by ANOVA. Further photosynthetic parameters and full numerical data, including statistical evaluation, are provided as Supporting Information Tables S2–S5. PGLP1, 2‐phosphoglycolate phosphatase 1.

To test if growth changes are due to altered photosynthesis, we measured Chla fluorescence and gas exchange parameters of plants grown under photorespiratory conditions. While PSI and PSII efficiencies and related parameters associated with photosynthetic light reactions did not significantly vary among the genotypes (Fig. S2; Table S2), light‐dependent net CO_2_ assimilation (*A*_N_) and stomatal conductance (*g*_s_) followed the growth pattern. Thus, *A*_N_ and *g*_s_ displayed a positive correlation with GC *PGLP1* expression (Fig. 2a–c). Transpiration rates (*E*), maximum photosynthetic rates (*A*_max_), and the slope of the light response curves (*α*_p_) followed this tendency (Table S3). Intracellular CO_2_ concentrations (*C*_i_) were only significantly decreased in the antisense lines, while intrinsic water‐use efficiency (iWUE) showed only minor alterations (Table S4). To check if photosynthetic stimulations rely on altered photorespiratory 2‐PG turnover in GC, we measured photosynthesis at three different O_2_ concentrations (3, 21, and 40%) to suppress or stimulate 2‐PG formation. At low photorespiratory flux requirements (3% O_2_), no significant changes in *A*_N_, *g*_s_, and the CO_2_ compensation points (*Γ*) were observed (Fig. 2d–f). However, at air O_2_ levels (21%), *A*_N_ was increased in overexpressor (*c*. 19%) and decreased in antisense lines (*c*. 13%), while *Γ* displayed inverse tendencies (*c*. 10% lower in overexpressors and *c*. 14% higher in antisense lines). Interestingly, *g*_s_, a parameter directly related to stomatal opening, was positively correlated with GC PGLP1 protein amounts and was higher (*c*. 29%) or lower (*c*. 16%) in the overexpressor and antisense lines, respectively (Fig. 2e; Table S5). The described patterns were similar at 40% O_2_, that is photorespiration‐stimulating conditions, but with stronger specification. In the overexpression lines, *A*_N_ and *g*_s_ were stimulated (*c*. 49 and 60%) and *Γ* decreased (*c*. 17%), while antisense repression caused a reduction in *A*_N_ and *g*_s_ (*c*. 33 and 27%) and a corresponding increase (*c*. 15%) in *Γ* (Fig. 2d–f). The calculated O_2_ sensitivity revealed overexpression lines were less and antisense lines more sensitive to O_2_ compared with the wild‐type (Fig. 2d, inlet). Finally, the slope of the *Γ*‐vs‐O_2_ concentration (*γ*), representing a measure of photorespiratory CO_2_ release, revealed that GC *PGLP1* overexpression caused a significant reduction, while antisense suppression caused an increase in photorespiratory CO_2_ losses (Fig. 2f, inlet).

### GC PGLP1 expression correlates with stomatal size, a morphological response that is inducible by external 2‐PG feeding to wild‐type Arabidopsis

**Fig. 3:**
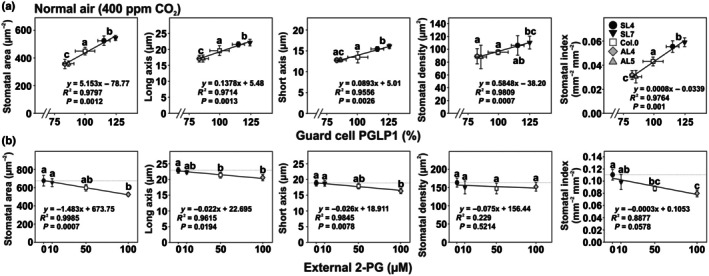
Characteristic stomatal parameters in Arabidopsis lines with GC‐specific *PGLP1* overexpression or antisense repression and Arabidopsis wild‐type plants grown with 2‐phosphoglycolate (2‐PG) supplementation. Plants were grown on soil in (a) air (400 ppm CO_2_) to stage 5.1 (Boyes *et al*., 2001) to determine stomata parameters. Displayed are correlation plots of selected stomatal parameters and GC‐specific PGLP1 protein expression in the transgenic lines and the wild‐type. Displayed are means ± SD (Σ 120 stomata per genotype were analyzed, from four biological replicates and 30 stomata per leaf). The corresponding high CO_2_ and all uncorrelated data are shown in Supporting information Fig. S3. The full numerical data is also provided as Table S6. Values that do not share the same letter are significantly different from each other as determined by ANOVA. (b) Arabidopsis wild‐type in air (400 ppm CO_2_) on half‐strength MS media supplemented with different 2‐PG concentrations (0, 10, 50 and 100 μM). After 2–3 wk stomatal parameters were determined by microscopic analysis as means ± SD (Σ 120 stomata per genotype, from four biological replicates and 30 stomata per leaf). Numbers in brackets indicate the reduction (in %, compared to control) with increased 2‐PG amounts: stomatal area (−3.2, −11.4 and −22.3%), stomatal length (−2.3, −5.9 and − 9.5%), stomatal width (−2.7, −3.0 and −11.5%), stomatal density (no significant changes), and stomatal index (−10.7, −20.4 and −27.7%). Full numerical data is provided in Supporting Information Table S7. Values that do not share the same letter are significantly different from each other as determined by ANOVA. PGLP1, 2‐phosphoglycolate phosphatase 1.

As GC PGLP1 amounts correlated with *g*_s_, we analyzed stomata count and size of all genotypes grown in air and elevated CO_2_ to compare the impact of photorespiratory and non‐photorespiratory conditions. As displayed in Fig. 3, stomata size, index, and density (significant only in the overexpressors) correlated with GC PGLP1 expression, as these parameters were increased and decreased in overexpression and antisense lines, respectively (Figs 3a, S3). These changes were photorespiration‐dependent as they were absent in high CO_2_‐grown plants (Fig. S3). Based on these findings, we hypothesized that altered GC PGLP1 expression and 2‐PG amounts could serve as a morphogenetic signal for stomatal development. To test this assumption, increasing 2‐PG concentrations (0, 10, 50, and 100 μM) were externally applied to Arabidopsis wild‐type plants during cultivation on agar plates. Interestingly, characteristic stomatal determinants showed a negative correlation with external 2‐PG application, as we measured gradually decreased length, width and smaller stomatal area and index compared to the control plants, lacking 2‐PG in the growth media. However, 2‐PG treatment had only minor effects on stomata density (Fig. 3c).

### Variations in whole‐leaf primary metabolism are restricted to soluble sugars, total amino acids, and organic acid contents

**Fig. 4:**
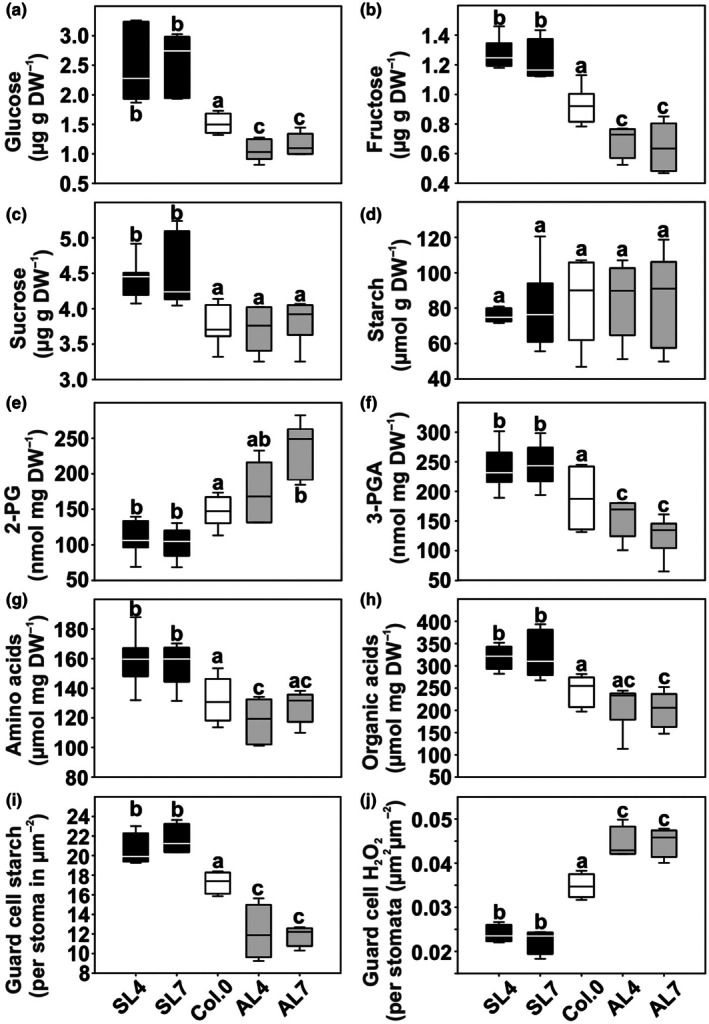
Selected metabolites in leaves and guard cells of Arabidopsis lines with GC‐specific *PGLP1* overexpression and antisense repression. Plants were grown in air (400 ppm CO_2_) to growth stage 5.1 (Boyes *et al*., 2001). Leaf material was harvested at end of the day (11 h illumination) and analyzed by (a–c) gas chromatography, (d) spectrophotometrically, LC‐MS/MS liquid chromatography coupled to tandem mass spectrometry (e–h) and microscopic (i, j) analysis. Values are means ± SD (*n* > 6). Values that do not share the same letter are significantly different from each other as determined by ANOVA. Full numerical data set of all metabolites are provided in Supporting Information Table S8. PGLP1, 2‐phosphoglycolate phosphatase 1; SL, sense line; AL, antisense line.

Because of the growth and photosynthetic responses of the transgenic lines, we quantified soluble sugars, starch, and 33 representatives of primary metabolism in leaves of all genotypes. Glucose and fructose levels were significantly higher in the overexpression lines and lower in the antisense lines. Sucrose was only higher in overexpressors (Fig. 4a–c), while transitory starch did not differ among genotypes (Fig. 4d). Further, leaf 2‐PG and NAD^+^ amounts showed a negative, while 3‐PGA showed a positive correlation with GC PGLP1 expression (Fig. 4e,f; Table S8). Among the other primary metabolites, we measured significant increases in glutamate, isoleucine, and isocitrate in the overexpression lines and significantly decreased arginine in the antisense lines. However, the calculation of total soluble sugars, amino, and organic acid contents revealed all to increase and decrease in the GC *PGLP1* overexpressors and antisense lines, respectively (Fig. 4g,h; Table S8).

### Manipulation of GC PGLP1 expression impacts on guard cell starch and H_2_O_2_ contents

Previous work suggested that GC starch and H_2_O_2_ amounts are involved in the energization and regulation of stomatal movements (Flütsch *et al*., 2020; Shi *et al*., 2024; Taylor *et al*., 2024). Hence, these parameters were measured under photorespiratory conditions, as no phenotypic or stomatal size variation were observed in high CO_2_‐grown plants. At one hand, we found a strong positive correlation between GC PGLP1 amounts and GC starch, which was significantly higher (*c*. 19–25%) in the overexpression and lower (*c*. 29–32%) in the antisense lines. On the other hand, H_2_O_2_ amounts were negatively correlated with GC PGLP1 expression, being lower (*c*. 31–36%) in overexpression and higher (*c*. 27–29%) in antisense lines compared to the wild‐type (Fig. 4i,j). Again, alterations in guard cell starch and H_2_O_2_ are photorespiration‐dependent as both were statistically invariant among the analyzed genotypes when grown under non‐photorespiratory conditions, that is, high CO_2_ (Table S8).

### Guard cell SBPase expression has no major impact on growth and photosynthesis

**Fig. 5:**
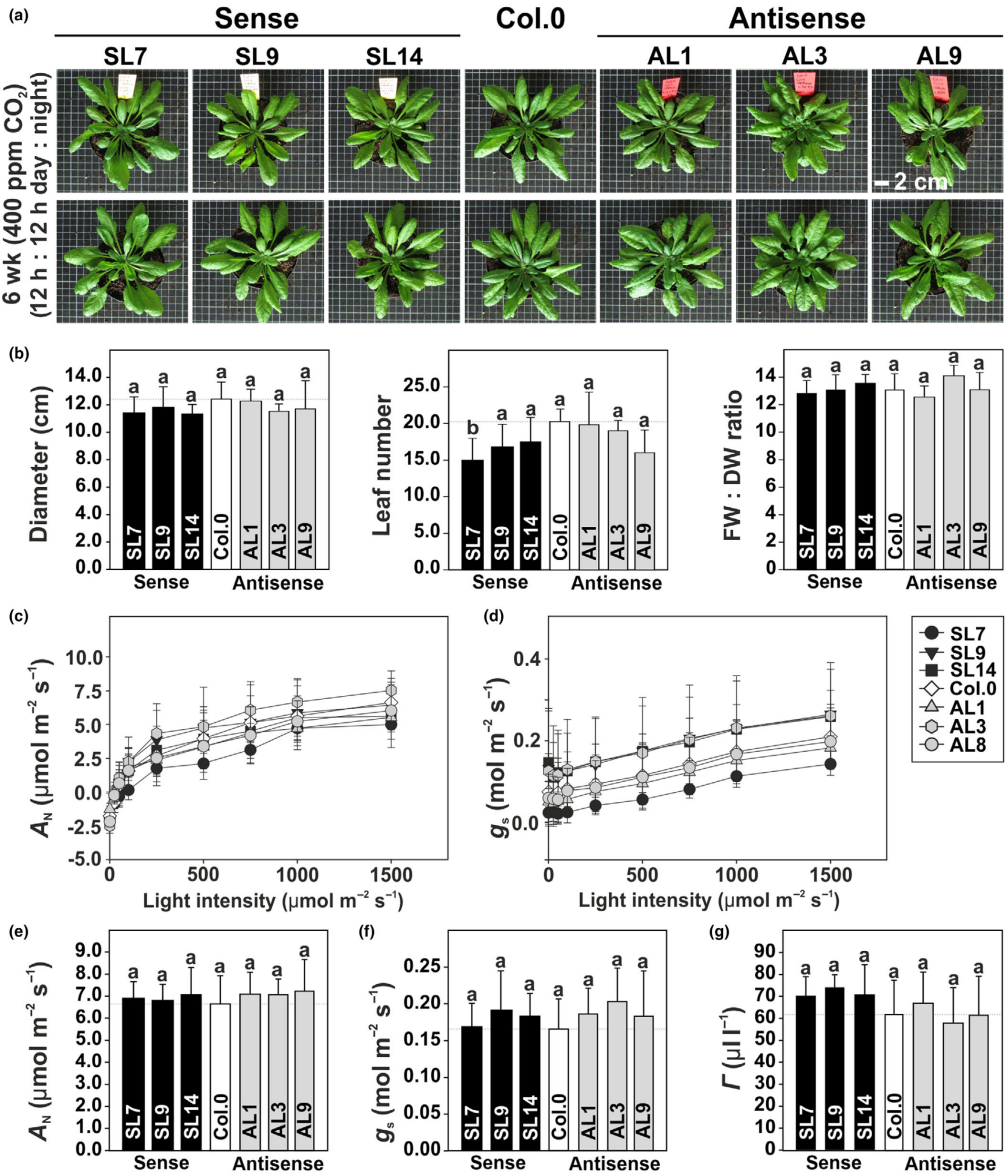
Phenotype, growth and photosynthesis of Arabidopsis lines with GC‐specific *SBPase* overexpression and antisense repression. (a) Representative photographs and (b) selected diagnostic growth parameters of the transgenic lines compared to the wild‐type following 6 wk growth under standard conditions in air (400 ppm CO_2_) with a 12 h : 12 h, day : night‐cycle. Selected parameters of the light response curves are given as follows: (c) Net CO_2_ uptake rates (*A*_N_) and, (d) stomatal conductance (*g*_s_) and functions of increasing light intensities. Selected parameters of the CO_2_ response curves are presented as follows: (e) *A*_N_, (f) *g*_s_, and (g) CO_2_ compensation points (*Γ*). Shown are means ± SD of at least six biological replicates. Values that do not share the same letter are significantly different from each other as determined by ANOVA. SBPase, sedoheptulose‐1,7‐bisphosphatase; SL, sense line; AL, antisense line.

Given that SBPase expression in leaves of various plant species positively correlates with growth and photosynthesis, and the fact that 2‐PG is a potent inhibitor of SBPase activity (Lefebvre *et al*., 2005; Ding *et al*., 2016; Driever *et al*., 2017; Flügel *et al*., 2017), we also manipulated the GC‐specific SBPase protein expression. In clear contrast to PGLP1 manipulations, we did not observe any significant impact on the visual phenotype and quantitative growth parameters of overexpression (+11.3–15.7% GC SBPase protein expression) and antisense (−12.6–22.48% GC SBPase protein expression) lines under the same growth conditions (Figs 5, S4). Furthermore, no consistent significant change was seen on selected photosynthetic parameters, including *A*_N_, *g*_s_, and *Γ* (Fig. 5), measured as functions of varying light and CO_2_.

## Discussion

**Fig. 6:**
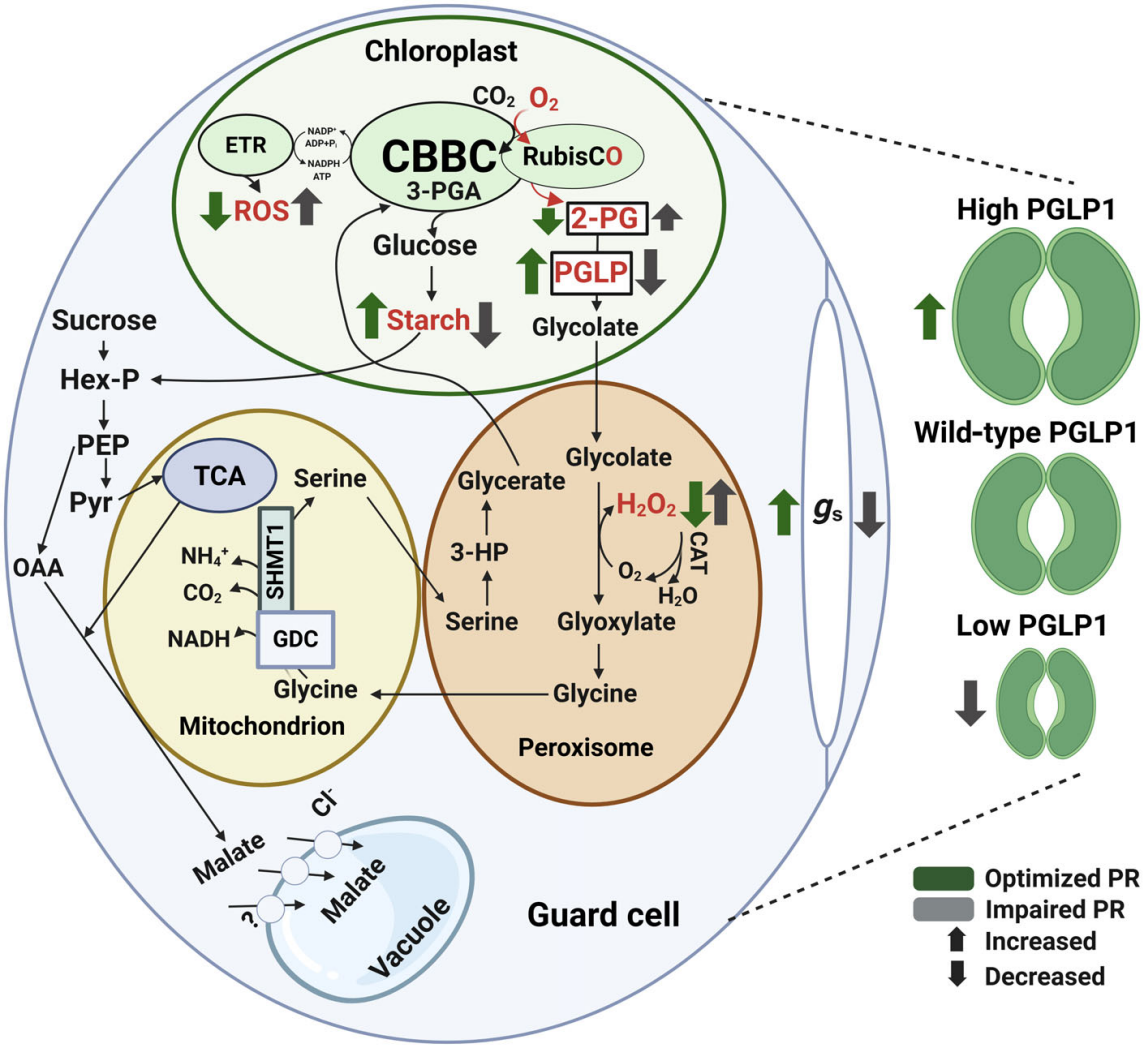
Model illustrating the impact of guard cell PGLP1 on stomatal behavior. Photorespiration is a highly compartmentalized process involving reactions in the chloroplast, peroxisome, mitochondrion, cytosol, and vacuole. While its roles and physiological implications are well established at the whole‐leaf level, particularly in the mesophyll, its function in specific tissues and cell types remains largely enigmatic. Guard cell‐specific manipulation of photorespiratory 2‐PG removal, achieved through upregulation or antisense repression of PGLP1, reveals photorespiration as a key component of guard cell metabolism as well as stomatal behavior and development in Arabidopsis. Mechanistically, we propose that changes in CO_2_ availability are sensed via the capacity of the photorespiratory flux, with the amount of the photorespiration‐specific entrance metabolite 2‐PG in chloroplasts serving as a key determinant. On the one hand, high chloroplast PGLP1 activity maintains low 2‐PG levels and alleviates negative impacts on CBBC performance, starch biosynthesis, and ROS accumulation, particularly H_2_O_2_, thereby supporting the carbohydrate and energy status required to drive stomatal movements. On the other hand, reduced PGLP1 activity leads to 2‐PG accumulation in chloroplasts, which slows the CBBC, decreases carbohydrate availability and metabolism, and promotes H_2_O_2_ accumulation. Consequently, stomata tend to remain more closed to prevent further damage to guard cells and the whole leaf, resulting from Rubisco oxygenation and impaired photorespiration. The figure was created with BioRender (https://BioRender.com/n08j5rq). 2‐PG, 2‐phosphoglycolate; 3‐PGA, 3‐phosphoglycerate; 3‐HP, 3‐hydroxypyruvate; CBBC, Calvin–Benson–Bassham cycle; CAT, catalase; ETR, electron transport chain; GDC, glycine decarboxylase; *g*_s_, stomatal conductance; Hex‐P, hexose phosphates; OAA, oxaloacetate; PEP, phosphoenolpyruvate; PGLP, 2‐PG phosphatase; Pyr, pyruvate; ROS, reactive oxygen species; SHMT1, serine‐hydroxymethyltransferase 1; TCA, tricarboxylic acid cycle.

Photorespiration is an unavoidable process in the primary C and N metabolism of plants, because it enables photosynthetic CO_2_ assimilation by detoxifying the Rubisco oxygenation product 2‐PG (Busch, 2020; Timm *et al*., 2025b). While the toxic effect of 2‐PG on plant metabolism is well established (Kelly & Latzko, 1976; Flügel *et al*., 2017; Li *et al*., 2019), it could also play a role as a low CO_2_‐sensing molecule in oxygenic phototrophs, as discussed to occur among cyanobacteria (Zhang *et al*., 2018). However, if 2‐PG plays such a role among plants remains uncertain to date. To address the question if 2‐PG is involved in CO_2_‐dependent stomata movements, we specifically manipulated the expression of the 2‐PG‐metabolizing enzyme PGLP1 in stomatal guard cells. This approach revealed a previously unrecognized function of PGLP1 or 2‐PG in coordinating GC metabolism with stomatal behavior (Fig. 6).

Although the observed changes in PGLP1 abundance in guard cells were comparably small compared to those observed in whole leaves (Fig. 1a; Flügel *et al*., 2017), they resulted in pronounced physiological effects. One possible explanation is that guard cells operate as highly sensitive regulatory units in which minor metabolic perturbations can strongly affect signaling pathways controlling stomatal aperture. Therefore, even modest changes in GC 2‐PG amounts could strongly inhibit various enzymatic reactions, including those involved in carbon assimilation and utilization, as discussed on a whole‐leaf basis. Further, it seems tempting to assume that GCs have a lower metabolic buffering capacity compared with MCs, and even small changes in enzyme levels could cause large local fluctuations of 2‐PG. However, our results provide a consistent picture: enhanced expression of PGLP1 in GC resulted in improved photosynthesis, higher stomatal conductance and enhanced growth, whereas antisense repression had opposite effects compared to wild‐type (Figs 1, 2). Importantly, growth stimulation depended on Rubisco‐mediated 2‐PG formation and its subsequent photorespiratory metabolization, as the transgenic lines were indistinguishable from the wild‐type under photorespiration‐suppressing conditions (Fig. 1; Table S1). Similarly, the differences in the photosynthetic parameters became larger when gas exchange measurements were done under high O_2_ conditions, stimulating photorespiration, while at lowered O_2_ levels they were virtually absent (Fig. 2). These findings suggest that GC metabolism is naturally constrained by PGLP1 activity under ambient, that is by the capacity of photorespiratory flux, and that increasing this capacity can benefit stomatal function through enhanced movement dynamics and energization (Fig. 6). This is particularly noteworthy given the ongoing debate regarding the extent to which GC perform photosynthesis and, perhaps, rely on active photorespiratory metabolism. Furthermore, GC PGLP1 limitation could be explained by specific features of these specialized cells, as they contain significantly fewer and smaller chloroplasts (Lawson *et al*., 2002; Lawson, 2009; Azoulay‐Shemer *et al*., 2015) and, thus, have generally naturally lower abundances of photorespiratory proteins. Until recently, the overall significance of photorespiratory metabolism in GC remained unclear due to the absence of guard cell‐specific transgenic approaches. The first GC‐specific manipulation of the mitochondrial photorespiratory enzyme glycine decarboxylase (GDC) provided evidence that these specialized cells are indeed capable of, and to some extent dependent on, active mitochondrial photorespiration (Sun *et al*., 2025). This conclusion is consistent with two recent proteomic studies on mitochondria isolated from GC and their specific ATP metabolism (Boussardon *et al*., 2025; Ditz *et al*., 2025) and earlier omics studies, showing the transcription and translation of the photorespiratory core cycle in GC (Yang *et al*., 2008; Wang *et al*., 2023).

The stimulation of growth, photosynthesis and stomatal conductance is consistent with earlier studies showing that GC‐specific modifications of different processes can positively influence *g*_s_ and *A*_N_ (Wang *et al*., 2014; Sun *et al*., 2025). Interestingly, the photorespiration‐specific results presented here, in conjunction with our earlier report, show that reprogrammed photorespiratory flux in different subcellular organelles has a clear and consistent impact on overall plant performance under ambient laboratory conditions. However, it remains unresolved whether higher photosynthetic rates arise directly from changes in *g*_s_ or whether PGLP1, and thereby photorespiratory flux, modifications in GC signals, and increased CO_2_ demand, which in turn prompts stomata to open more widely to support mesophyll photosynthesis and facilitate a higher energy status of the cells. Notably, the latter interpretation aligns with reports of photorespiratory optimizations, mainly PGLP1 and GDC overexpression, at the whole‐leaf level, which also resulted in higher stomatal conductance (Timm *et al*., 2012a, 2015, 2025a; Flügel *et al*., 2017). Given that the previously used *ST*‐*LSI* promoter is not fully mesophyll‐specific and also drives expression in GC, it could well be that the observed responses are also caused by expression changes of both enzymes in GC. Taken together, these observations support the hypothesis that photorespiratory flux capacity, mediated through reinforcement or alleviation of negative feedback on carbon utilization, could serve as a key determinant for sensing and translating changes in external (*C*_a_) and internal (*C*_i_) CO_2_ availability. More specifically, and supported by our findings, we suggest that chloroplastidal 2‐PG could mechanistically serve as signaling metabolite translating altered photorespiratory fluxes in response to changes in CO_2_ availability. The ultimate readout of such a mechanism could be shifts in the availability of photosynthates and other biomolecules at the whole‐leaf level. Indeed, the metabolite profiles of the transgenic models support this hypothesis, given GC PGLP1 protein expression positively correlated with soluble sugars, as well as the total amino acid and organic acid contents (Fig. 4; Table S8). It should also be noted that changes in the GC photorespiratory flux, that is the chloroplastidal 2‐PG amount, seem to be causative for optimized photosynthesis and growth, rather than alleviated negative feedback inhibition of the central CBB cycle enzyme SBPase as GC overexpression of the latter did not result in similar physiological responses (Figs 5, S4). Interestingly, recent metabolomic and modeling studies have highlighted the remarkable metabolic flexibility of GC and the central role of carbon metabolism in regulating stomatal movements (Sprent *et al*., 2024; Zait *et al*., 2025). These studies suggest that GC metabolism operates as an integrated network in which relatively small perturbations in specific pathways can result in substantial physiological responses. In this context, alterations in photorespiratory enzymes such as PGLP1 may influence GC metabolism through various mechanisms. First, changes in 2‐PG levels could modulate CBB cycle activity, likely SBPase‐independent, and thereby redirect carbon flux toward glycolysis and organic acid metabolism, processes that are closely linked to the generation of osmolytes required for stomatal movements. Second, photorespiration is tightly connected to cellular redox metabolism and energy balance, which are key determinants of ion transport and proton pump activity in GC. Finally, photorespiratory metabolism may influence the apoplastic environment through changes in ROS production or organic acid fluxes, thereby affecting signaling processes that regulate stomatal aperture. Together, these considerations support the idea that photorespiration represents an important metabolic component of GC regulatory networks controlling stomatal function.

GC starch availability and metabolism were reported to be key determinants of GC energization and their rapid movements to acclimate to environmental fluctuations (Santelia & Lunn, 2017; Flütsch *et al*., 2020; Zhang *et al*., 2025). Although transitory starch stocks underwent no significant changes on the whole‐leaf basis among our transgenic plants, GC starch accumulation correlated with GC PGLP expression in the transgenic lines (Figs 1, 4; Table S6). Hence, starch availability and turnover seem to be, at least to some extent, controlled by GC photorespiration. Similar alterations were found before, that is, lowered 2‐PG levels due to *PGLP1* overexpression stimulated starch synthesis and elevated 2‐PG levels due to *PGLP1* antisense repressed starch accumulation on the whole‐leaf basis (Flügel *et al*., 2017). However, if the different starch amounts in GC are a direct effect of altered GC photorespiration or its reprogrammed mesophyll metabolism and carbon import thereof, it has to be analyzed at higher resolution in the future. Nevertheless, increased GC starch seems to be a general response of GC overexpression of photorespiratory enzymes as similar observations were made on corresponding GDC manipulations (Sun *et al*., 2025).

In addition to starch, the GC‐localized amounts of H_2_O_2_, another central player in stomatal regulation (Li *et al*., 2020; da Silva *et al*., 2024; Shi *et al*., 2024; Taylor *et al*., 2024), were inversely correlated with GC PGLP1 expression in air (Fig. 4j). The exact origin of the altered H_2_O_2_ in our lines remains an open question. Photorespiratory H_2_O_2_ production seems unlikely at first look, as flux scaling would predict opposite trends between overexpression and antisense lines; however, the lack of H_2_O_2_ variations in high CO_2_ suggests that the photorespiratory flux contributes to the overall H_2_O_2_ pool (Table S8). Alternative sources, such as imbalances in mitochondrial or chloroplastidial electron transport and thereby produced H_2_O_2_, also lack support from our fluorescence and rETR(I) data. By contrast, NADPH oxidase activity emerges as a plausible candidate, potentially explaining the observed discrepancy between H_2_O_2_ accumulation and stomatal aperture. An additional possibility is that changes in ROS detoxification capacity contribute to the altered H_2_O_2_ profiles. Hence, in addition to the observed metabolic alterations, our findings highlight a potential role of H_2_O_2_ in linking GC PGLP1 activity and its potential role in CO_2_ sensing to stomatal function. Typically, low concentrations of H_2_O_2_ promote stomatal opening via nuclear localization of KIN10 and subsequent induction of BAM1 and AMY3, driving starch degradation (Li *et al*., 2020; Shi *et al*., 2024). At higher concentrations, however, H_2_O_2_ triggers stomatal closure through activation of Ca^2+^ channels and the anion channel SLAC1, largely mediated by NADPH oxidases (RBOHs) (Chater *et al*., 2015; Sierla *et al*., 2016). Photorespiration represents a major source of hydrogen peroxide in plant cells due to the activity of glycolate oxidase in peroxisomes (Foyer *et al*., 2009). Alterations in PGLP1 activity GC may therefore influence the metabolic flux through the photorespiratory pathway and consequently affect intracellular H_2_O_2_ production. Because ROS act as central signaling components controlling stomatal movements (Sierla *et al*., 2016; Shi *et al*., 2024), photorespiratory H_2_O_2_ could contribute to the modulation of GC signaling pathways. In addition, intracellular ROS pools are known to interact with NADPH oxidase (RBOH)‐dependent oxidative bursts that regulate stomatal responses. It is therefore conceivable that changes in GC photorespiration alter the redox environment and thereby influence RBOH‐mediated ROS production. Further, antioxidant enzymes such as catalase, which play a central role in detoxifying photorespiratory H_2_O_2_, have been reported to interact functionally with RBOH‐dependent signaling pathways (Sierla *et al*., 2016; Li *et al*., 2022). Together, these observations support the idea that photorespiratory metabolism may contribute to stomatal regulation by modulating ROS signaling networks in guard cells.

As discussed earlier, stomatal conductance could be directly or indirectly related to the reprogrammed GC metabolism via GC‐specific PGLP1 manipulation. However, in contrast to the manipulation of GC‐specific photorespiration due to GDC expression changes (Sun *et al*., 2025), GC‐specific PGLP1 manipulations also affected stomatal morphology. Specifically, GC PGLP1 abundance positively correlated with stomatal size and, to some extent, density (Figs 3, S3; Table S6). These morphological adaptations can certainly contribute to altered stomatal conductance, as maximal conductance (*g*_smax_) largely depends on stomatal size and density (Franks & Beerling, 2009a,b). Thus, it seems reasonable to assume that enhanced PGLP1 activity, and thereby more efficient degradation of GC 2‐PG, leading to lower steady‐state GC 2‐PG levels, could underlie the observed changes in stomatal size. Given that the direct quantification of GC 2‐PG remains technically challenging, we tested whether exogenous 2‐PG influences stomatal traits in Arabidopsis wild‐type. Indeed, increasing external 2‐PG supply gradually reduced stomatal dimensions (Fig. 4c; Table S7), supporting the hypothesis that optimal GC PGLP1 activity, through 2‐PG detoxification, is fundamental for maintaining proper GC and stomatal morphology. This observation also provides evidence that not changed amounts of GC PGLP1, but directly its substrate 2‐PG, serves as a signaling molecule. This finding, thus, could be taken as a direct hint for its role in the signaling of different CO_2_ levels not only via impacting on GC movements, but also GC development in plants. This statement is in line with evolutionary observations that low CO_2_ (high 2‐PG) selected for smaller, while high CO_2_ (low 2‐PG) for larger stomata (Franks *et al*., 2009; Drake *et al*., 2013; Inoue & Kinoshita, 2017).

Overall, our findings strongly support the view that GC photorespiration, including GC‐specific 2‐PG degradation, is a fundamental component of stomatal metabolism and behavior. This metabolic framework may serve as the basis for coordinating environmental variations that strongly influence photorespiratory fluxes with GC behavior and mesophyll metabolism. By modulating 2‐PG detoxification and ROS homeostasis within GC, PGLP1 influences both stomatal size and conductance, thereby regulating CO_2_ availability for photosynthesis. Together, these results highlight GC photorespiration as an underappreciated target for enhancing crop productivity, particularly under conditions where photorespiration is active.

## Competing interests

None declared.

## Author contributions

ST conceived and supervised the project. HS, IT and ST designed the research. NS and ST performed cloning procedures and established the transgenic lines. HS and IT performed the research. HS, IT, JK, TL, MH and ST analyzed the data. MH provided experimental equipment and tools. HS and ST wrote the article, with additions and revisions from JK, TL and MH. All authors have read and approved the final version of the manuscript.

## Disclaimer

The New Phytologist Foundation remains neutral with regard to jurisdictional claims in maps and in any institutional affiliations.

## Supporting information


**Fig. S1** Generation and verification of Arabidopsis lines with GC‐specific PGLP1 overexpression and antisense repression.
**Fig. S2** Chlorophyll fluorescence parameters of Arabidopsis lines with GC‐specific PGLP1 overexpression and antisense repression grown in air.
**Fig. S3** Stomata parameters of Arabidopsis lines with GC‐specific PGLP1 overexpression and antisense repression grown in air and high CO_2_.
**Fig. S4** Generation and verification of Arabidopsis lines with GC‐specific SBPase overexpression and antisense repression.
**Table S1** Quantitative growth data of Arabidopsis lines with GC‐specific PGLP1 overexpression and antisense repression in air and high CO_2_.
**Table S2** Numeric data of Chl fluorescence measurements of Arabidopsis lines with GC‐specific PGLP1 overexpression and antisense repression grown in air.
**Table S3** Calculated parameters from gas exchange light response curves of Arabidopsis lines with GC‐specific PGLP1 overexpression and antisense repression grown in air.
**Table S4** Numeric data of light response curves of Arabidopsis lines with GC‐specific PGLP1 overexpression and antisense repression grown in air.
**Table S5** Numeric data of O_2_‐dependent CO_2_ response curves of Arabidopsis lines with GC‐specific PGLP1 overexpression and antisense repression grown in air.
**Table S6** Numeric data of stomata parameters of Arabidopsis lines with GC‐specific PGLP1 overexpression and antisense repression grown in air and high CO_2_.
**Table S7** Numeric data of stomata parameters of Arabidopsis wild‐type plants grown on different 2‐PG concentrations in air.
**Table S8** Numeric data of the metabolite analysis of Arabidopsis lines with GC‐specific PGLP1 overexpression and antisense repression grown in air.
**Table S9** Primers used for PCR amplification.
Please note: Wiley is not responsible for the content or functionality of any Supporting Information supplied by the authors. Any queries (other than missing material) should be directed to the *New Phytologist* Central Office.

## Data Availability

The data supporting the findings of this study are presented as Figs 1, 2, 3, 4, 5 included in the main text and in the Supporting Information (Figs S1–S4; Tables S1–S9) associated with this article. Plasmids and transgenic plants generated in this study will be made available upon request to the corresponding author.
