# Supplementary material for: Guard cell photorespiration controls stomata behavior and development
Source: New Phytol. 2026 Mar 26;250(5):3182–96. doi: 10.1111/nph.71137 (PMC13150294; doi:10.1111/nph.71137)
Supplement: Supplementary file 1 — Fig. S1 Generation and verification of Arabidopsis lines with GC‐specific PGLP1 overexpression and antisense repression. Fig. S2 Chlorophyll fluorescence parameters of Arabidopsis lines with GC‐specific PGLP1 overexpression and antisense repression grown in air. Fig. S3 Stomata parameters of Arabidopsis lines with GC‐specific PGLP1 overexpression and antisense repression grown in air and high CO2. Fig. S4 Generation and verification of Arabidopsis lines with GC‐specific SBPase overexpression and antisense repression. Table S1 Quantitative growth data of Arabidopsis lines with GC‐specific PGLP1 overexpression and antisense repression in air and high CO2. Table S2 Numeric data of Chl fluorescence measurements of Arabidopsis lines with GC‐specific PGLP1 overexpression and antisense repression grown in air. Table S3 Calculated parameters from gas exchange light response curves of Arabidopsis lines with GC‐specific PGLP1 overexpression and antisense repression grown in air. Table S4 Numeric data of light response curves of Arabidopsis lines with GC‐specific PGLP1 overexpression and antisense repression grown in air. Table S5 Numeric data of O2‐dependent CO2 response curves of Arabidopsis lines with GC‐specific PGLP1 overexpression and antisense repression grown in air. Table S6 Numeric data of stomata parameters of Arabidopsis lines with GC‐specific PGLP1 overexpression and antisense repression grown in air and high CO2. Table S7 Numeric data of stomata parameters of Arabidopsis wild‐type plants grown on different 2‐PG concentrations in air. Table S8 Numeric data of the metabolite analysis of Arabidopsis lines with GC‐specific PGLP1 overexpression and antisense repression grown in air. Table S9 Primers used for PCR amplification. Please note: Wiley is not responsible for the content or functionality of any Supporting Information supplied by the authors. Any queries (other than missing material) should be directed to the New Phytologist Central Office. [file NPH-250-3182-s001.pdf]

New Phytologist

New Phytologist Supporting Information

Article title: **Guard cell photorespiration controls stomata behavior and development**
Authors: Hu Sun, Inken Thiemann, Nils Schmidt, Johannes Kromdijk, Tracy Lawson, Martin Hagemann, Stefan Timm
Article acceptance date: 09 March 2026

The following Supporting Information is available for this article:

Fig. S1 Generation and verification of Arabidopsis lines with GC-specific *PGLP1* overexpression and antisense repression.

Fig. S2 Chlorophyll fluorescence parameters of Arabidopsis lines with GC-specific *PGLP1* overexpression and antisense repression grown in air.

Fig. S3 Stomata parameters of Arabidopsis lines with GC-specific *PGLP1* overexpression and antisense repression grown in air and high CO_2.

Fig. S4 Generation and verification of Arabidopsis lines with GC-specific *SBPase* overexpression and antisense repression.

Table S1 Quantitative growth data of Arabidopsis lines with GC-specific *PGLP1* overexpression and antisense repression in air and high CO_2.

Table S2 Numeric data of chlorophyll fluorescence measurements of Arabidopsis lines with GC-specific *PGLP1* overexpression and antisense repression grown in air.

Table S3 Calculated parameters from gas exchange light response curves of Arabidopsis lines with GC-specific *PGLP1* overexpression and antisense repression grown in air.

Table S4 Numeric data of light response curves of Arabidopsis lines with GC-specific *PGLP1* overexpression and antisense repression grown in air.

Table S5 Numeric data of O_2-dependent CO_2 response curves of Arabidopsis lines with GC-specific *PGLP1* overexpression and antisense repression grown in air.

Table S6 Numeric data of stomata parameters of Arabidopsis lines with GC-specific *PGLP1* overexpression and antisense repression grown in air and high CO_2.

Table S7 Numeric data of stomata parameters of Arabidopsis wildtype plants grown on different 2-PG concentrations in air.

Table S8 Numeric data of the metabolite analysis of Arabidopsis lines with GC-specific *PGLP1* overexpression and antisense repression grown in air.

Table S9 Primers used for PCR amplification.

Fig. S1 Generation and verification of Arabidopsis lines with GC-specific *PGLP1* overexpression and antisense repression. Overview of the GC-specific *PGLP1* **(A)** overexpression (sense) and **(B)** antisense repression constructs. **(C)** Genotypic validation of the transformed constructs into the genome of transgenic **(C1)** overexpression and **(C2)** antisense lines and the corresponding loading control **(C3)** via PCR. **(D)** RT-PCR validation of diagnostic fragments of the transgenic *SlPGLP1* transcript in **(D1)** overexpression and **(D2)** antisense lines, in comparison with signals of the **(D3)** constitutively expressed 40S ribosomal protein *S16* gene as control. **(E)** Densitometric analysis of PGLP1 protein expression analyzed via immunoblotting experiments of protein extracts from **(E1)** GC and **(E2)** MC, using a specific antibody against PGLP1 and GDC-H or RbcL as loading controls. Note, control immunoblots were essentially developed from the same membrane as used for PGLP1. Given are means ± SD of (E) three independent immunoblots. Values that do not share the same letter are significantly different from each other as determined by ANOVA.

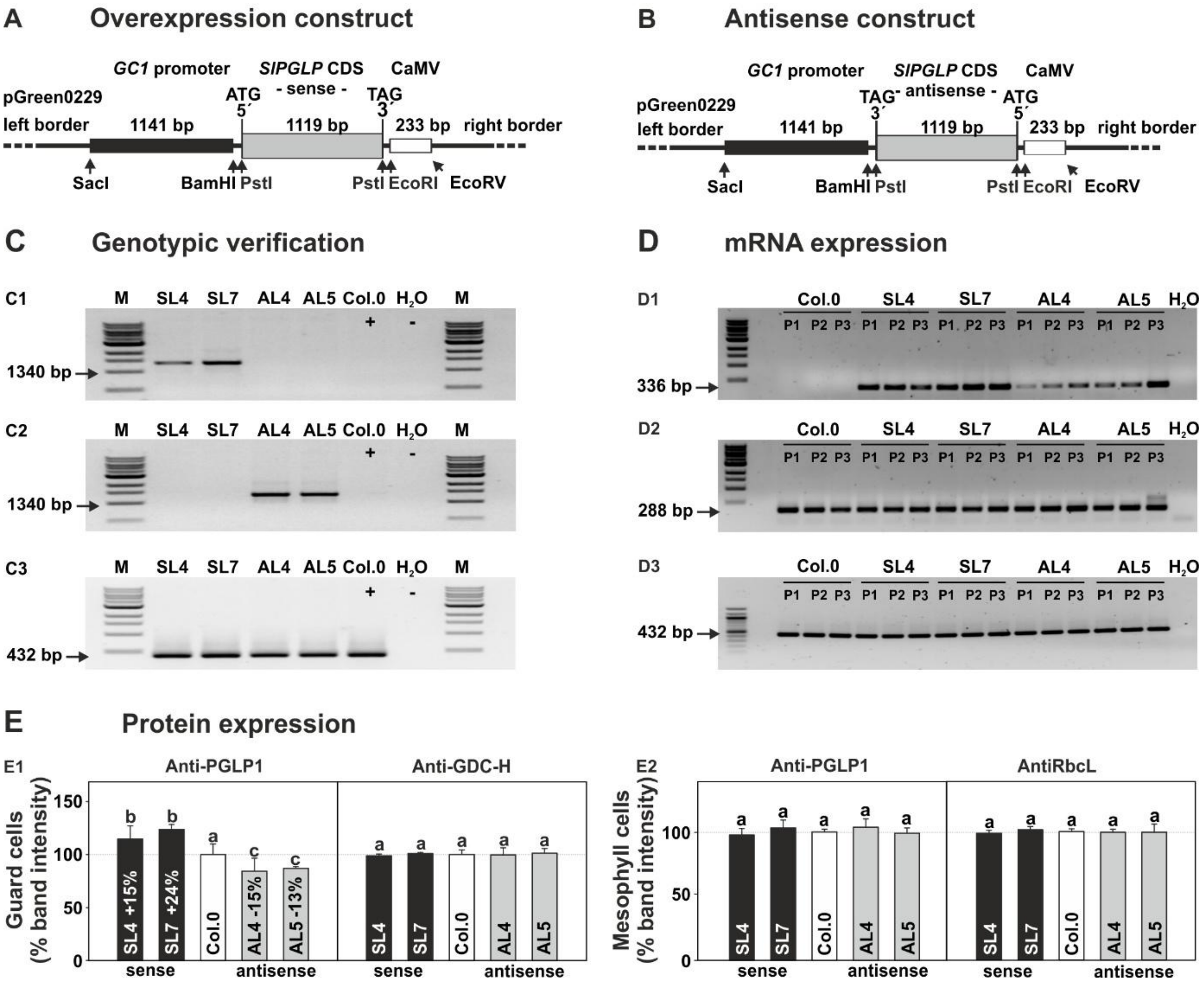
A Overexpression construct
GC1 promoter
SlPGLP CDS - sense -
CaMV
pGreen0229 left border
1141 bp
ATG 5´
1119 bp
TAG 3´
233 bp
right border
SacI
BamHI PstI
PstI EcoRI
EcoRV
B Antisense construct
GC1 promoter
SlPGLP CDS - antisense -
CaMV
pGreen0229 left border
1141 bp
TAG 3´
1119 bp
ATG 5´
233 bp
right border
SacI
BamHI PstI
PstI EcoRI
EcoRV
C Genotypic verification
C1 M SL4 SL7 AL4 AL5 Col.0 H2O M
+ -
1340 bp
C2 M SL4 SL7 AL4 AL5 Col.0 H2O M
+ -
1340 bp
C3 M SL4 SL7 AL4 AL5 Col.0 H2O M
+ -
432 bp
D mRNA expression
D1 Col.0 SL4 SL7 AL4 AL5 H2O
P1 P2 P3 P1 P2 P3 P1 P2 P3 P1 P2 P3 P1 P2 P3
336 bp
D2 Col.0 SL4 SL7 AL4 AL5 H2O
P1 P2 P3 P1 P2 P3 P1 P2 P3 P1 P2 P3 P1 P2 P3
288 bp
D3 Col.0 SL4 SL7 AL4 AL5 H2O
P1 P2 P3 P1 P2 P3 P1 P2 P3 P1 P2 P3 P1 P2 P3
432 bp
E Protein expression
E1 Anti-PGLP1 Anti-GDC-H
Guard cells (% band intensity)
0 50 100 150
SL4 +15% SL7 +24% Col.0 AL4 -15% AL5 -13%
b b a c c
SL4 SL7 Col.0 AL4 AL5
a a a a a
sense antisense sense antisense
E2 Anti-PGLP1 AntiRbcL
Mesophyll cells (% band intensity)
0 50 100
SL4 SL7 Col.0 AL4 AL5
a a a a a
SL4 SL7 Col.0 AL4 AL5
a a a a a
sense antisense sense antisense

Fig. S2 Chlorophyll fluorescence parameters of Arabidopsis lines with GC-specific PGLP1 overexpression and antisense repression grown in air. Given are selected PSII and PSI characteristics of all genotypes grown under standard growth conditions to stage 5.1 (Boyes et al. 2001). Displayed are: **(A)** Maximum efficiency of PSII (F_v/F_m) and maximum oxidizable P700 (Pm) from dark adapted plants; **(B)** photosynthetic efficiency curves of PSII (Y[II]) and PSI (Y[I]); **(C)** relative electron transport rates of PSII (rETR II) and PSI (rETR I); **(D)** non-photochemical quenching of PSII (NPQ) and cyclic electron flow around PSI (CET); and, **(E)** acceptor (Y[NA]) and donor (Y[ND]) side limitation of PSI. Shown are means ± SD of at least 6 biological replicates. Values that do not share the same letter are significantly different from each other as determined by ANOVA. Note, lack of significance letters in B to E is explained due to lack of statistical differences.

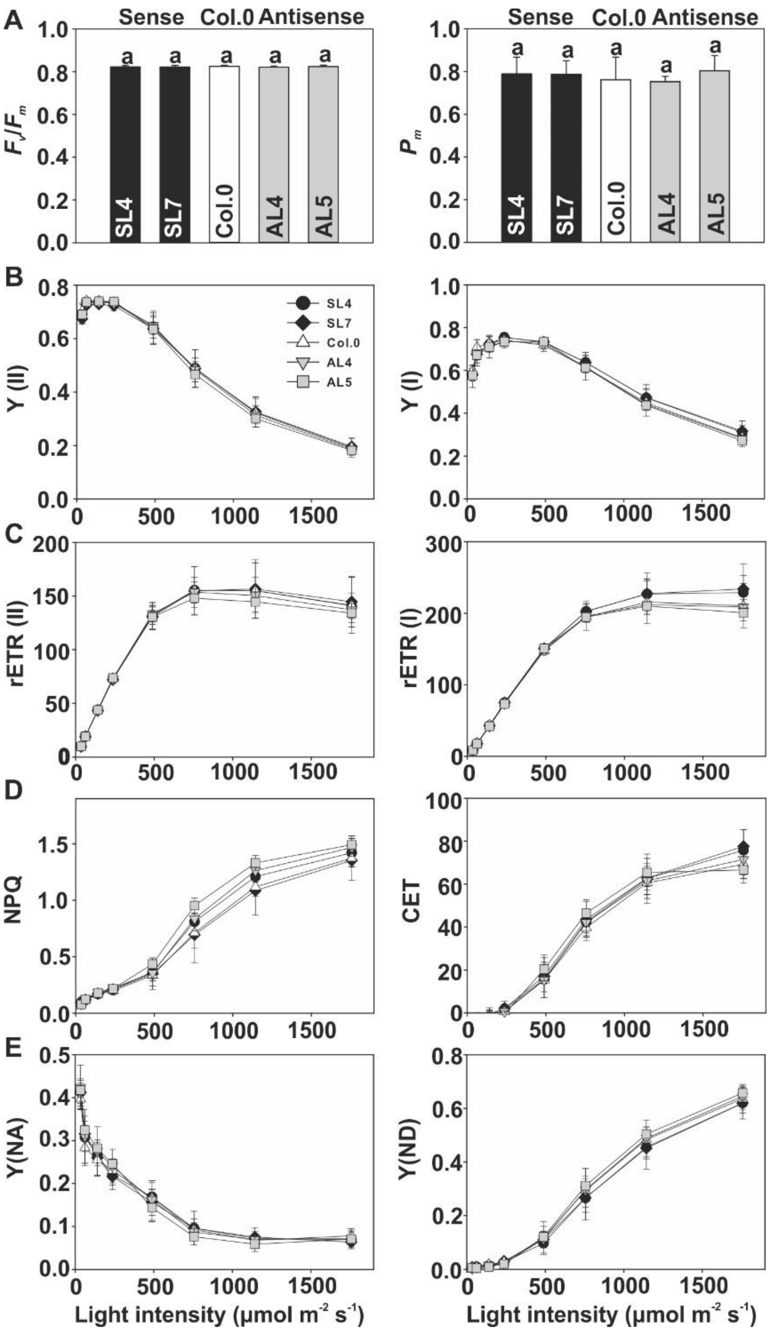

A
Sense
Col.0
Antisense
F_v/F_m
P_m
a
SL4
SL7
Col.0
AL4
AL5
B
Y (II)
Y (I)
SL4
SL7
Col.0
AL4
AL5
C
rETR (II)
rETR (I)
D
NPQ
CET
E
Y(NA)
Y(ND)
Light intensity (μmol m^{-2} s^{-1})

Fig. S3 Stomata parameters of Arabidopsis lines with GC-specific *PGLP1* overexpression and antisense repression grown in air and high CO_2. Plants were grown in air (400 ppm) and high CO_2 (3000 ppm) to stage 5.1 (Boyes et al. 2001) to determine stomata parameters. Given are means ± SD (Σ 120 stomata per genotype was analyzed, from 4 biological replicates and 30 stomata per leaf) for: **(A)** stomata area, **(B)** stomata length, **(C)** stomata width, **(D)** stomatal density, and **(E)** stomatal index. Black bars show air and grey bars high CO_2 values. Values that do not share the same letter are significantly different from each other as determined by ANOVA. For full numerical data see Supp. Table S6.

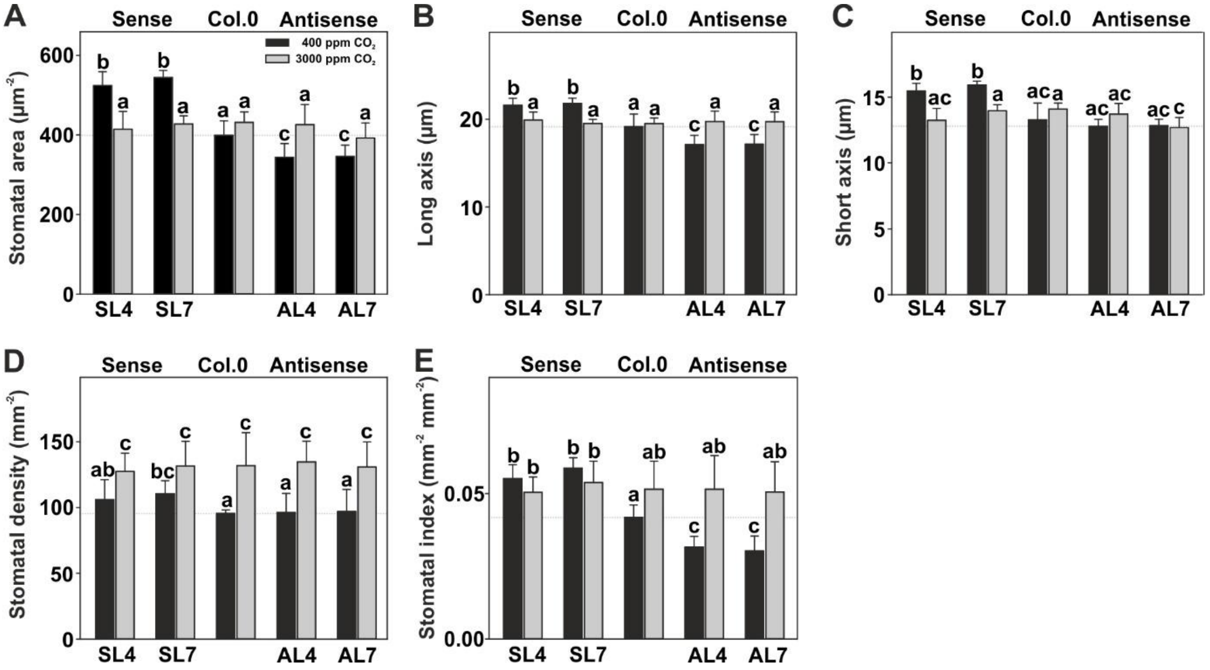

Fig. S4 Generation and verification of Arabidopsis lines with GC-specific *SBPase* overexpression and antisense repression. Overview of the GC specific *AtSBPase* **(A)** overexpression (sense) and **(B)** antisense repression constructs. **(C)** Genotypic validation of the transformed constructs into the genome of transgenic **(C1)** overexpression and **(C2)** antisense lines and the corresponding loading control **(C3)** via PCR. **(D)** Densitometric analysis of SBPase (upper panel) and PGLP1 (lower panel) protein expression analyzed via immunoblotting experiments of protein extracts from GC (left) and MC (right), using a specific antibody against SBPase and PGLP1 as loading control. Note, control immunoblots were essentially developed from the same membrane as used for SBPase. Given are means ± SD of three independent immunoblots. Values that do not share the same letter are significantly different from each other as determined by ANOVA.

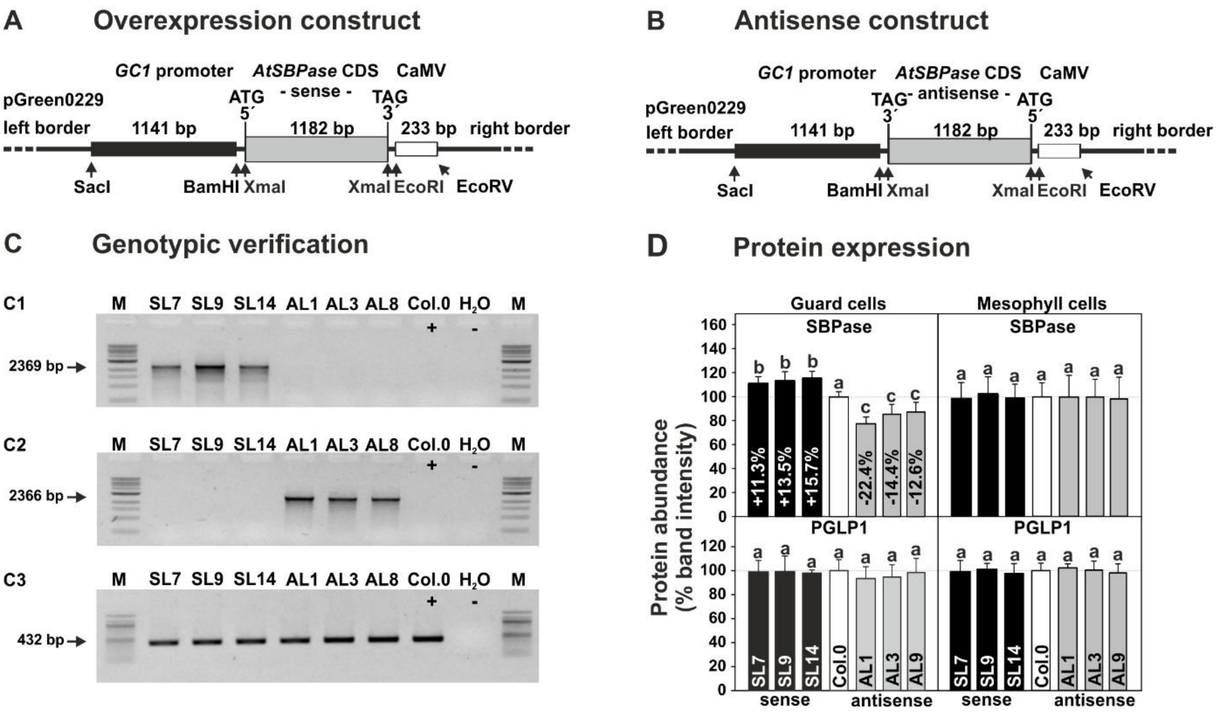

Table S1 Quantitative growth data of Arabidopsis lines with GC-specific PGLP1 overexpression and antisense repression in air and high CO2.Plants were grown under environmental controlled conditions in air (LC – low carbon; 400 ppm CO2; 6 weeks) and high CO2 (HC; 3000 ppm CO2; 4 weeks) to growth stage 5.1 (Boyes et al. 2001). Selected quantitative growth parameters are means ± SD of at least six biological replicates. Values that do not share the same letter (in bold) are significantly different from each other as determined by ANOVA analysis.

	**Genotype**	**Parameters**			
		Fresh weight (g)	Dry weight (g)	Diameter (cm)	Leaf number
LC	SL4	**2.71 ± 0.43[b]**	**0.216 ± 0.027[b]**	**13.87 ± 0.66[b]**	**26.00 ± 2.01[b]**
	SL7	**2.77 ± 0.27[b]**	**0.222 ± 0.016[b]**	**14.20 ± 0.55[b]**	**25.83 ± 2.23[b]**
	Col.0	1.93 ± 0.33[a]	0.162 ± 00.26[a]	12.55 ± 0.77[a]	19.67 ± 2.50[a]
	AL4	**1.47 ± 0.22[c]**	**0.125 ± 0.015[c]**	**10.95 ± 0.91[c]**	**16.83 ± 1.75[c]**
	AL5	**1.35 ± 0.27[c]**	**0.108 ± 0.021[c]**	**11.27 ± 0.91[c]**	**17.67 ± 1.75[c]**
HC	SL4	1.04 ± 0.13[a]	0.117 ± 0.016[a]	9.17 ± 0.62[a]	17.50 ± 0.55[a]
	SL7	1.05 ± 0.21[a]	0.122 ± 0.017[a]	9.47 ± 0.96[a]	17.83 ± 0.75[a]
	Col.0	1.12 ± 0.24[a]	0.130 ± 0.021[a]	9.33 ± 1.02[a]	17.67 ± 0.82[a]
	AL4	1.16 ± 0.14[a]	0.116 ± 0.021[a]	9.60 ± 0.95[a]	17.33 ± 1.21[a]
	AL5	1.16 ± 0.19[a]	0.120 ± 0.019[a]	9.23 ± 0.94[a]	17.50 ± 0.84[a]

Table S2 Numeric data of chlorophyll fluorescence measurements of Arabidopsis lines with GC-specific PGLP1 overexpression and antisense repression grown in air. Plants were grown under environmental controlled conditions in air (400 ppm CO2) to growth stage 5.1 (Boyes et al. 2001). Light response curves were measured from low-to-high light (34, 62, 141, 237, 489, 756, 1144, and 1757 µmol m-2 s-1) following 10 min of dark adaptation. Shown are: photosynthetic efficiency of PSII (Y[II]) and PSI (Y[I]), relative electron transport rates of PSII (rETR II) and PSI (rETR I), non-photochemical quenching of PSI (NPQ), cyclic electron transport around PSI (CET) and acceptor (Y[NA]) and donor (Y[ND]) side limitation of PSI parameters. Values are means ± SD (n = 6). Values that do not share the same letter are significantly different from each other as determined by ANOVA.

Parameter	Genotype	Light intensity (µmol protons m^{-2} s^{-1})							
		34	62	141	237	489	756	1144	1757
Y(II)	SL4	0.69 ± 0.04[a]	0.74 ± 0.02[a]	0.74 ± 0.01[a]	0.73 ± 0.02[a]	0.64 ± 0.02[a]	0.49 ± 0.04[a]	0.32 ± 0.03[a]	0.19 ± 0.02[a]
	SL7	0.70 ±0.02[a]	0.73 ±0.01[a]	0.73 ±0.01[a]	0.72 ±0.02[a]	0.64 ±0.02[a]	0.49 ±0.07[a]	0.33 ±0.06[a]	0.20 ±0.03[a]
	Col.0	0.69 ± 0.03[a]	0.74 ± 0.01[a]	0.74 ± 0.01[a]	0.73 ± 0.01[a]	0.65 ± 0.01[a]	0.49 ± 0.07[a]	0.32 ± 0.05[a]	0.19 ± 0.04[a]
	AL4	0.69 ± 0.02[a]	0.74 ± 0.01[a]	0.74 ± 0.01[a]	0.73± 0.01[a]	0.64± 0.01[a]	0.48 ± 0.03[a]	0.31 ± 0.02[a]	0.19 ± 0.01[a]
	AL5	0.69 ± 0.02[a]	0.74 ± 0.01[a]	0.74 ± 0.01[a]	0.74 ± 0.01[a]	0.64 ± 0.01[a]	0.47 ± 0.02[a]	0.30 ± 0.02[a]	0.18 ± 0.01[a]
Y(I)	SL4	0.10 ± 0.05[a]	0.12 ± 0.02[a]	0.17 ± 0.03[a]	0.21 ± 0.03[a]	0.37 ± 0.07[a]	0.81 ± 0.07[a]	1.21 ± 0.13[a]	1.42 ± 0.13[a]
	SL7	0.09 ± 0.02[a]	0.12 ± 0.01[a]	0.17 ± 0.01[a]	0.21 ± 0.02[a]	0.35 ± 0.14[a]	0.69 ± 0.25[a]	1.09 ± 0.22[a]	1.35 ± 0.17[a]
	Col.0	0.08 ± 0.04[a]	0.12 ± 0.01[a]	0.17 ± 0.01[a]	0.20 ± 0.04[a]	0.34 ± 0.10[a]	0.71 ± 0.14[a]	1.12 ± 0.08[a]	1.37 ± 0.07[a]
	AL4	0.08 ± 0.01[a]	0.13 ± 0.01[a]	0.18 ± 0.02[a]	0.22 ± 0.02[a]	0.36 ± 0.07[a]	0.84 ± 0.11[a]	1.26 ± 0.10[a]	1.47 ± 0.08[a]
	AL5	0.08 ± 0.01[a]	0.12 ± 0.01[a]	0.18 ± 0.01[a]	0.22 ± 0.02[a]	0.44 ± 0.05[a]	0.95 ± 0.07[a]	1.33 ± 0.07[a]	1.49 ± 0.08[a]
rETR (II)	SL4	9.9 ± 0.5[a]	19.2 ± 0.5[a]	43.7 ± 0.8[a]	72.9 ± 1.7[a]	132.3 ± 8.7[a]	155.5 ± 12.0[a]	155.1 ± 12.5[a]	140.9 ± 11.9[a]
	SL7	9.6 ± 0.3[a]	18.9 ± 0.3[a]	43.1 ± 0.7[a]	72.0 ± 1.8[a]	130.9 ± 12.2[a]	154.8 ± 22.5[a]	156.6 ± 27.1[a]	144.7 ± 23.4[a]
	Col.0	10.0 ± 0.4[a]	19.3 ± 0.4[a]	43.8 ± 0.7[a]	73.1 ± 1.2[a]	132.0 ± 12.4[a]	155.2 ± 22.1[a]	155.0 ± 26.0[a]	141.3 ± 26.0[a]
	AL4	9.9 ± 0.2[a]	19.1 ± 0.2[a]	43.6 ± 0.3[a]	73.0 ± 0.6[a]	133.5 ± 6.3[a]	153.7 ± 9.3[a]	150.4 ± 9.8[a]	137.1 ± 8.2[a]
	AL5	9.9 ± 0.2[a]	19.2 ± 0.2[a]	43.8 ± 0.4[a]	73.6 ± 0.6[a]	147.9 ± 4.5[a]	148.0 ± 8.9[a]	144.5 ± 9.6[a]	134.1 ± 8.8[a]
	SL4	8.3 ± 0.3[a]	17.8 ± 0.8[a]	42.7 ± 1.3[a]	75.1 ± 1.7[a]	150.7 ± 4.0[a]	202.6 ± 9.8[a]	226.8 ± 20.8[a]	228.9 ± 24.0[a]

rETR (I)	SL7	8.3 ± 0.4[a]	17.6 ± 1.1[a]	42.9 ± 2.4[a]	75.0 ± 2.3[a]	150.5± 2.7[a]	202.4 ± 14.9[a]	227.3 ± 28.8[a]	233.9 ± 35.2[a]
	Col.0	8.5 ± 0.3[a]	18.4 ± 0.9[a]	43.0 ± 1.8[a]	73.9 ± 1.8[a]	147.3 ± 5.8[a]	194.9 ± 18.9[a]	215.5 ± 30.0[a]	210.6 ± 30.9[a]
	AL4	8.2 ± 0.7[a]	17.8 ± 1.6[a]	42.0 ± 3.0[a]	73.6 ± 3.5[a]	148.8 ± 4.7[a]	195.8 ± 7.4[a]	211.9 ± 9.4[a]	208.6 ± 10.8[a]
	AL5	8.2 ± 0.3[a]	17.5 ± 0.6[a]	42.0 ± 0.9[a]	73.0 ± 1.1[a]	150.9 ± 2.8[a]	194.5 ± 7.2[a]	209.9 ± 11.6[a]	200.6 ± 11.2[a]
NPQ	SL4	0.10 ± 0.05[a]	0.12 ± 0.02[a]	0.17 ± 0.03[a]	0.21 ± 0.03[a]	0.37 ± 0.07[a]	0.81 ± 0.07[a]	1.21 ± 0.13[a]	1.42 ± 0.13[a]
	SL7	0.09 ± 0.02[a]	0.12 ± 0.01[a]	0.17 ± 0.01[a]	0.21 ± 0.02[a]	0.35 ± 0.14[a]	0.69 ± 0.25[a]	1.09 ± 0.17[a]	1.35 ± 0.17[a]
	Col.0	0.09 ± 0.04[a]	0.12 ± 0.01[a]	0.17 ± 0.02[a]	0.20 ± 0.04[a]	0.34 ± 0.10[a]	0.71 ± 0.14[a]	1.12 ± 0.07[a]	1.37 ± 0.07[a]
	AL4	0.08 ± 0.01[a]	0.13 ± 0.01[a]	0.18 ± 0.02[a]	0.22 ± 0.02[a]	0.36 ± 0.07[a]	0.84 ± 0.11[a]	1.26 ± 0.10[a]	1.47 ± 0.08[a]
	AL5	0.08 ± 0.01[a]	0.12 ± 0.01[a]	0.18 ± 0.01[a]	0.22 ± 0.02[a]	0.44 ± 0.05[a]	0.95 ± 0.07[a]	1.33 ± 0.07[a]	1.49 ± 0.08[a]
CET	SL4	- 1.6 ± 0.5[a]	- 1.3 ± 0.4[a]	- 0.9 ± 1.4[a]	1.8 ± 2.0[a]	15.6 ± 2.1[a]	42.3 ± 5.0[a]	62.5 ± 11.4[a]	75.7 ± 9.5[a]
	SL7	- 1.4 ± 0.5[a]	- 1.5 ± 1.1[a]	- 0.4 ± 2.8[a]	2.2 ± 3.2[a]	17.1 ± 9.9[a]	43.5 ± 8.2[a]	62.9 ± 7.8[a]	77.6 ± 7.8[a]
	Col.0	- 1.5 ± 0.5[a]	- 0.9 ± 0.8[a]	- 0.8 ± 1.8[a]	0.7 ± 1.6[a]	15.3 ± 8.2[a]	39.7 ± 6.0[a]	60.5 ± 6.7[a]	69.3 ± 6.7[a]
	AL4	- 1.7 ± 0.7[a]	- 1.4 ± 1.5[a]	- 1.6 ± 2.9[a]	0.6 ± 3.5[a]	15.4 ± 5.5[a]	42.1 ± 6.2[a]	61.5 ± 7.4[a]	71.5 ± 7.4[a]
	AL5	- 1.6 ± 0.4[a]	- 1.7 ± 0.6[a]	- 1.9 ± 0.8[a]	- 0.5 ± 1.4[a]	20.4 ± 5.5[a]	46.5 ± 6.2[a]	65.4 ± 6.1[a]	66.6 ± 6.1[a]
Y (NA)	SL4	0.41 ± 0.03[a]	0.31 ± 0.03[a]	0.27 ± 0.02[a]	0.22 ± 0.02[a]	0.17 ± 0.02[a]	0.10 ± 0.01[a]	0.07 ± 0.01[a]	0.07 ± 0.01[a]
	SL7	0.41 ± 0.03[a]	0.32 ± 0.04[a]	0.26 ± 0.04[a]	0.22 ± 0.03[a]	0.16 ± 0.05[a]	0.10 ± 0.04[a]	0.08 ± 0.02[a]	0.06 ± 0.02[a]
	Col.0	0.40 ± 0.02[a]	0.28 ± 0.04[a]	0.26 ± 0.04[a]	0.23 ± 0.02[a]	0.17 ± 0.04[a]	0.09 ± 0.03[a]	0.07 ± 0.01[a]	0.08 ± 0.02[a]
	AL4	0.42 ± 0.05[a]	0.31 ± 0.06[a]	0.28 ± 0.06[a]	0.24 ± 0.04[a]	0.16 ± 0.02[a]	0.09 ± 0.02[a]	0.07 ± 0.02[a]	0.07 ± 0.02[a]
	AL5	0.42 ± 0.02[a]	0.32 ± 0.02[a]	0.28 ± 0.01[a]	0.25 ± 0.01[a]	0.14 ± 0.03[a]	0.08 ± 0.02[a]	0.06 ± 0.02[a]	0.07 ± 0.02[a]
Y (ND)	SL4	0.005 ± 0.005[a]	0.009 ± 0.008[a]	0.014 ± 0.005[a]	0.026 ± 0.010[a]	0.097 ± 0.017[a]	0.266 ± 0.034[a]	0.456 ± 0.037[a]	0.620 ± 0.024[a]
	SL7	0.008 ± 0.006[a]	0.009 ± 0.006[a]	0.015 ± 0.005[a]	0.030 ± 0.009[a]	0.111 ± 0.050[a]	0.266 ± 0.082[a]	0.452 ± 0.079[a]	0.620 ± 0.059[a]
	Col.0	0.006 ± 0.003[a]	0.009 ± 0.008[a]	0.018 ± 0.009[a]	0.027 ± 0.008[a]	0.116 ± 0.062[a]	0.294 ± 0.082[a]	0.483 ± 0.073[a]	0.636 ± 0.053[a]
	AL4	0.003 ± 0.003[a]	0.008 ± 0.006[a]	0.015 ± 0.010[a]	0.020 ± 0.004[a]	0.113 ± 0.028[a]	0.296 ± 0.027[a]	0.489 ± 0.022[a]	0.645 ± 0.013[a]
	AL5	0.005 ± 0.003[a]	0.004 ± 0.004[a]	0.009 ± 0.004[a]	0.020 ± 0.006[a]	0.121 ± 0.021[a]	0.311 ± 0.027[a]	0.504 ± 0.026[a]	0.657 ± 0.026[a]

Table S3 Calculated parameters from gas exchange light response curves of Arabidopsis lines with GC-specific *PGLP1* overexpression and antisense repression grown in air. Estimations of the maximum photosynthetic rate (A_{max}) and initial slopes of the gas exchange light response curves (α_p) measured by from the transgenic lines in comparison with the wildtype grown under air conditions to stage 5.1 [43]. Given are means ± SD (n = 6). Values that do not share the same letter (in bold) are significantly different from each other as determined by ANOVA. Light response curves are shown in Fig. 2 and the corresponding full numerical data are provided as Supp. Table S4.

Genotype	Parameter	
	A_{max}	α_p
**SL4**	**9.76 ± 0.56^{b}**	**0.098 ± 0.012^{b}**
**SL7**	**9.62 ± 0.51^{b}**	**0.095 ± 0.007^{b}**
**Col.0**	7.64 ± 0.79^{a}	0.078 ± 0.009^{a}
**AL4**	**5.83 ± 0.73^{c}**	**0.059 ± 0.009^{c}**
**AL5**	**5.97 ± 0.81^{c}**	**0.056 ± 0.012^{c}**

Table S4 Numeric data of light response curves of Arabidopsis lines with GC-specific *PGLP1* overexpression and antisense repression grown in air. Plants were grown under environmental controlled conditions in air (400 ppm CO_2) to growth stage 5.1 (Boyes et al. 2001). Light response curves were measured from high-to-low light (1600, 1200, 800, 400, 200, 100, 50, 25, and 0 µmol m^{-2} s^{-1}) following 10 min of light adaptation at 1000 µmol m^{-2} s^{-1}. Given are: net CO_2 assimilation rate (A_N), stomatal conductance (g_s), intracellular CO_2 concentration (C_i), transpiration rate (E); and intrinsic water use efficiency (*WUE*). Values are means ± SD (n = 6). Values that do not share the same letter (in bold) are significantly different from each other as determined by ANOVA. A_N and g_s graphs (in grey) are displayed in Fig. 2A-B and deduced values of A_{max} and α_p are provided in Supp. Table S3.

		Light intensity (µmol protons m^{-2} s^{-1})								
	**Genotype**	**0**	**25**	**50**	**100**	**200**	**400**	**800**	**1200**	**1600**
	SL4	- 1.46 ± 0.34^{a}	0.63 ± 0.15^{a}	**1.74 ± 0.35^{b}**	**3.85 ± 0.16^{b}**	**6.73 ± 0.18^{b}**	**8.21 ± 0.44^{b}**	**9.09 ± 0.59^{b}**	**9.89 ± 0.92^{b}**	**9.98 ± 0.70^{b}**
A_N	SL7	- 1.12 ± 0.51^{a}	0.50 ±0.20^{a}	**1.75 ± 0.23^{b}**	**3.98 ± 0.32^{b}**	**6.61 ± 0.21^{b}**	**8.11 ± 0.22^{b}**	**8.73 ± 0.45^{b}**	**9.04 ± 0.39^{b}**	**9.41 ± 45^{b}**
(µmol CO_2 m^{-2} s^{-1})	Col.0	- 1.16 ± 0.13^{a}	0.32 ± 0.28^{a}	1.37 ± 0.21^{ab}	3.07 ± 0.48^{a}	5.44 ± 0.59^{a}	6.64 ± 0.61^{a}	7.09 ± 0.63^{a}	7.33 ± 0.94^{a}	7.71 ± 0.83^{a}
	AL4	- 1.05 ± 0.27^{a}	0.22 ± 0.27^{a}	1.09 ± 0.30^{a}	**2.32 ± 0.51^{c}**	**4.09 ± 0.92^{c}**	**4.96 ± 0.76^{c}**	**5.45 ± 0.91^{c}**	**5.75 ± 0.73^{c}**	**5.58 ± 0.87^{c}**
	AL5	- 0.79 ± 0.12^{a}	0.44 ± 0.16^{a}	1.19 ± 0.24^{a}	**2.75 ± 0.07^{ac}**	**4.70 ± 0.32^{c}**	**5.57 ± 0.22^{c}**	**6.11 ± 0.15^{c}**	**6.14 ± 0.29^{c}**	**6.07 ± 0.42^{c}**
	SL4	**0.14 ± 0.02^{b}**	**0.15 ± 0.01^{b}**	**0.17 ± 0.01^{b}**	**0.19 ± 0.01^{b}**	**0.21 ± 0.01^{b}**	**0.22 ± 0.01^{b}**	**0.23 ± 0.01^{b}**	**0.24 ± 0.02^{b}**	**0.25 ± 0.02^{b}**
g_s	SL7	**0.14 ± 0.01^{b}**	**0.15 ± 0.01^{b}**	**0.17 ± 0.01^{b}**	**0.19 ± 0.01^{b}**	**0.21 ± 0.001^{b}**	**0.23 ± 0.01^{b}**	**0.24 ± 0.01^{b}**	**0.25 ± 0.01^{b}**	**0.26 ± 0.01^{b}**
(mol m^{-2} s^{-1})	Col.0	0.11 ± 0.01^{a}	0.12 ± 0.01^{a}	0.13 ± 0.01^{a}	0.14 ± 0.01^{a}	0.16 ± 0.01^{a}	0.17 ± 0.01^{a}	0.18 ± 0.01^{a}	0.18 ± 0.01^{a}	0.20 ± 0.02^{a}
	AL4	**0.06 ± 0.03^{c}**	**0.07 ± 0.03^{c}**	**0.08 ± 0.03^{c}**	**0.09 ± 0.03^{c}**	**0.10 ± 0.03^{c}**	**0.11 ± 0.03^{c}**	**0.12 ± 0.03^{c}**	**0.12 ± 0.03^{c}**	**0.12 ± 0.03^{c}**
	AL5	**0.06 ± 0.03^{c}**	**0.07 ± 0.03^{c}**	**0.08 ± 0.04^{c}**	**0.09 ± 0.04^{c}**	**0.10 ± 0.04^{c}**	**0.11 ± 0.04^{c}**	**0.12 ± 0.04^{c}**	**0.12 ± 0.04^{c}**	**0.11 ± 0.04^{c}**
	SL4	410.28 ± 4.19^{a}	385.91 ± 1.98^{a}	375.27 ± 3.59^{a}	357.67 ± 2.91^{a}	335.88 ± 2.97^{a}	326.51 ± 2.99^{a}	321.41 ± 3.10^{a}	317.63 ± 2.19^{a}	318.80 ± 4.54^{a}
C_i	SL7	406.35 ± 7.02^{a}	386.47 ± 2.51^{a}	374.32 ± 2.53^{a}	356.10 ± 3.67^{a}	337.13 ± 1.18^{a}	328.65 ± 1.21^{a}	326.50 ± 1.52^{a}	326.25 ± 4.43^{a}	326.63 ± 3.17^{a}
(µmol)	Col.0	410.63 ± 2.53^{a}	387.93 ± 4.53^{a}	374.42 ± 4.59^{a}	355.95 ± 7.51^{a}	333.11 ± 8.74^{a}	324.60 ± 10.5^{ab}	322.24 ± 8.43^{a}	322.33 ± 8.36^{a}	322.44 ± 8.12^{a}

	AL4	**440.38 ± 28.36[b]**	389.11 ± 1.81[a]	361.91 ± 15.70[a]	339.99 ± 19.20[a]	**314.24 ± 22.27[ab]**	**304.97 ± 16.42[b]**	**301.35 ± 15.38[b]**	**293.94 ± 12.02[b]**	**294.26 ± 16.87[b]**
	AL5	421.74± 18.59[ab]	375.85 ± 11.85[b]	361.24 ± 9.06[a]	331.90 ± 20.54[a]	**306.50 ± 20.04[b]**	**300.77 ± 18.05[b]**	**293.83 ± 20.65[b]**	**289.47 ± 23.77[b]**	**283.35 ± 27.54[b]**
	SL4	**1.49 ± 0.10[b]**	**1.61 ± 0.09[b]**	**1.76 ± 0.08[b]**	**1.95 ± 0.09[b]**	**2.14 ± 0.12[b]**	**2.35 ± 0.15[b]**	**2.64 ± 0.18[b]**	**2.64 ± 0.20[b]**	**3.27 ± 0.24[b]**
E	SL7	**1.58 ± 0.07[b]**	**1.71 ± 0.08[b]**	**1.88 ± 0.07[b]**	**2.10 ± 0.07[b]**	**2.32 ± 0.07[b]**	**2.55 ± 0.09[b]**	**2.85 ± 0.11[b]**	**3.13 ± 0.11[b]**	**3.46 ± 0.10[b]**
(μmol H_2O m^{-2} s^{-1})	Col.0	1.18 ± 0.09[a]	1.27 ± 0.09[a]	1.38 ± 0.09[a]	1.53 ± 0.07[a]	1.68 ± 0.07[a]	1.85 ± 0.08[a]	2.06 ± 0.10[a]	2.28 ± 0.12[a]	2.56 ± 0.16[a]
	AL4	**0.81 ± 0.38[c]**	**0.89 ± 0.43[c]**	**1.02 ± 0.46[c]**	**1.18 ± 0.49[c]**	**1.34 ± 0.48[c]**	**1.52 ± 0.51[c]**	**1.69 ± 0.53[c]**	**1.83 ± 0.55[c]**	**1.94 ± 0.53[c]**
	AL5	**0.83 ± 0.36[c]**	**0.92 ± 0.37[c]**	**1.06 ± 0.38[c]**	**1.23 ± 0.39[c]**	**1.41 ± 0.37[c]**	**1.57 ± 0.35[c]**	**1.72 ± 0.39[c]**	**1.79 ± 0.41[c]**	**1.80 ± 0.51[c]**
	SL4	- 0.99 ± 0.0.28[a]	0.39 ± 0.10[a]	0.99 ± 0.18[a]	1.98 ± 0.13[a]	3.15 ± 0.11[a]	3.51 ± 0.21[a]	3.45 ± 0.16[s]	3.36 ± 0.20[a]	3.06 ± 0.11[a]
WUE	SL7	- 0.72 ± 0.0.37[a]	0.29 ± 0.11[a]	0.93 ± 0.13[a]	1.89 ± 0.15[a]	2.85 ± 0.07[a]	3.18 ± 0.07[a]	3.07 ± 0.10[b]	**2.89 ± 0.07[b]**	**2.72 ± 0.08[b]**
(μmol CO_2 mol^{-1}	Col.0	- 0.98 ± 0.13[a]	0.25 ± 0.22[a]	1.00 ± 0.20[a]	2.00 ± 0.30[a]	3.23 ± 0.30[a]	3.59 ± 0.44[a]	3.44 ± 0.22[a]	3.21 ± 0.29[ab]	3.01 ± 0.23[a]
	AL4	- 1.95 ± 1.16[a]	0.31 ± 0.25[a]	1.39 ± 0.55[a]	2.39 ± 0.69[a]	3.45 ± 0.85[a]	3.56 ± 0.70[a]	3.43 ± 0.62[sb]	3.29 ± 0.61[ab]	3.04 ± 0.70[ab]
	AL5	- 1.25 ± 0.79[a]	0.61 ± 0.42[a]	1.25 ± 0.37[a]	2.48 ± 0.83[a]	3.55 ± 0.76[a]	3.69 ± 0.68[a]	3.72 ± 0.69[ab]	3.62 ± 0.68[ab]	**3.58 ± 0.68[d]**

Table S5 Numeric data of O_2-dependent CO_2 response curves of Arabidopsis lines with GC-specific *PGLP1* overexpression and antisense repression grown in air. Plants were grown under environmental controlled conditions in air (400 ppm CO_2) to growth stage 5.1 (Boyes et al. 2001). CO_2 response curves (400, 0, 50, 100, 200, 300, 400 ppm CO_2) were measured following 10 min of light adaptation (1000 µmol m^{-2} s^{-1}) at 3, 21 and 40% O_2. Given are: net CO_2 assimilation rate (A_N), stomatal conductance (g_s), intracellular CO_2 concentration (C_i), transpiration rate (E), intrinsic water use efficiency (WUE) and net CO_2 compensation points (Γ). Values are means ± SD (n = 6). Values that do not share the same letter are significantly different (in bold) from each other as determined by ANOVA. Selected parameters (in grey) are displayed in Fig. 3D-F of the main manuscript.

O_2	Genotype	A_N µmol CO_2 m^{-2} s^{-1}	g_s mol m^{-2} s^{-1}	C_i µmol	E µmol H_2O m^{-2} s^{-1}	*WUE* µmol CO_2 mol^{-1} H_2O	Γ µL L^{-1}
3%	SL4	10.61 ± 1.40[a]	0.18 ± 0.025[a]	284.8 ± 17.6[a]	4.18 ± 0.30[a]	59.4 ± 10.5[a]	15.2 ± 2.72[a]
	SL7	10.00 ± 0.85[a]	0.15 ± 0.049[a]	258.1 ± 37.4[a]	3.96 ± 1.01[a]	73.9 ± 23.1[a]	13.3 ± 5.10[a]
	Col.0	9.55 ± 0.84[a]	0.13 ± 0.048[a]	232.9 ± 38.8[a]	3.72 ± 1.11[a]	79.5 ± 23.4[a]	14.8 ± 4.23[a]
	AL4	9.96 ± 0.99[a]	0.17 ± 0.051[a]	277.2 ± 21.5[a]	4.36 ± 1.61[a]	62.1 ± 13.6[a]	13.9 ± 3.9[a]
	AL5	9.82 ± 0.85[a]	0.14 ± 0.033[a]	251.9 ± 32.9[a]	3.97 ± 0.79[a]	77.1 ± 20.5[a]	16.7 ± 5.9[a]
21%	SL4	**9.38 ± 0.32[b]**	**0.27 ± 0.054[b]**	327.8 ± 10.7[a]	**3.52 ± 0.33[b]**	39.3 ± 7.0[a]	**57.5 ± 6.17[a]**
	SL7	**9.62 ± 0.94[b]**	**0.24 ± 0.042[b]**	316.4 ± 9.4[a]	**3.33 ± 0.53[b]**	42.9 ± 6.3[a]	**58.6 ± 3.60[a]**
	Col.0	7.83 ± 0.48[a]	0.19 ± 0.013[a]	324.7 ± 13.4[a]	2.58 ± 0.19[a]	38.9 ± 8.8[a]	65.3 ± 2.77[a]
	AL4	**6.63 ± 0.81[c]**	**0.16 ± 0.026[c]**	329.2 ± 12.3[a]	**2.00 ± 0.31[c]**	36.0 ± 8.0[a]	**75.1 ± 2.81[a]**
	AL5	**6.82 ± 0.48[c]**	**0.15 ± 0.025[c]**	325.5 ± 15.1[a]	**1.95 ± 0.29[c]**	37.9 ± 9.8[a]	**74.5 ± 3.19[a]**
40%	SL4	**7.38 ± 1.50[b]**	**0.21 ± 0.055[b]**	317.5 ± 23.2[a]	**4.43 ± 1.05[b]**	39.6 ± 13.9[a]	**108.5 ± 10.38[a]**
	SL7	**7.45 ± 1.83[b]**	**0.19 ± 0.069[b]**	310.3 ± 33.1[a]	**4.64 ± 1.27[b]**	42.7 ± 19.7[a]	**105.0 ± 11.81[a]**
	Col.0	4.93 ± 1.23[a]	0.12 ± 0.036[a]	311.6 ± 15.5[a]	3.27 ± 0.72[a]	42.6 ± 9.7[a]	130.7 ± 6.18[a]
	AL4	**3.09 ± 0.46[c]**	**0.09 ± 0.032[c]**	330.6 ± 10.5[a]	**2.51 ± 0.54[c]**	31.4 ± 6.2[a]	**152.0 ± 7.32[a]**
	AL5	**3.30 ± 0.39[c]**	**0.09 ± 0.010[c]**	316.7 ± 8.3[a]	**2.63 ± 0.30[c]**	38.2 ± 5.3[a]	**151.1 ± 8.03[a]**

Table S6 Numeric data of stomata parameters of Arabidopsis lines with GC-specific *PGLP1* overexpression and antisense repression grown in air and high CO_2.

Plant were grown in air (400 ppm) and high CO_2 (3000 ppm) to stage 5.1 (Boyes et al. 2001) to determine stomata parameters, including stomata area, stomata length, stomata width, stomatal density, and stomatal index. Given are means ± SD (Σ 120 stomata per genotype was analyzed, from 4 biological replicates and 30 stomata per leaf). Values that do not share the same letter (in bold) are significantly different from each other as determined by ANOVA. This data relates to the correlation plots shown in Fig. 3A-J of the main manuscript and the comparative graphs are displayed as Supp. Fig S3.

CO_2 concentration	Genotype	Parameter				
		Stomatal area (μm^{-2})	Long axis (μm)	Short axis (μm)	Stomatal density (mm^{-2})	Stomatal index ($mm^{2}mm^{-2}$)
400 ppm	**SL4**	**524.25 ± 34.31[b]**	**21.59 ± 0.79[b]**	**15.45 ± 0.58[b]**	105.99 ± 15.22[ab]	**0.055 ± 0.0048[b]**
	**SL7**	**544.33 ± 17.49[b]**	**22.14 ± 1.02[b]**	**16.10 ± 0.53[b]**	**110.46 ± 9.99[bc]**	**0.059 ± 0.0036[b]**
	**Col.0**	422.72 ± 36.76[a]	19.63 ± 0.91[a]	13.69 ± 0.62[ac]	95.59 ± 2.70[a]	0.043 ± 0.0037[a]
	**AL4**	**356.11 ± 33.03[c]**	**17.11 ± 1.05[c]**	12.77 ± 0.53[ac]	89.49 ± 11.93[a]	**0.032 ± 0.0037[c]**
	**AL5**	**358.69 ± 18.52[c]**	**17.15 ± 1.09[c]**	12.82 ± 0.48[ac]	88.04 ± 18.25[a]	**0.030 ± 0.0052[c]**
3000 ppm	**SL4**	414.06 ± 44.98[a]	19.91 ± 0.91[a]	13.22 ± 0.90[ac]	**127.47 ± 13.85[c]**	**0.051 ± 0.0053[b]**
	**SL7**	427.47 ± 20.41[a]	19.51 ± 0.47[a]	13.94 ± 0.46[a]	**131.60 ± 18.81[c]**	**0.054 ± 0.0073[b]**
	**Col.0**	431.47 ± 26.13[a]	19.50 ± 0.64[a]	14.08 ± 0.45[a]	**131.92 ± 25.02[c]**	0.052 ± 0.0096[ab]
	**AL4**	425.66 ± 50.81[a]	19.74 ± 1.19[a]	13.69 ± 0.81[ac]	**134.70 ± 15.71[c]**	0.052 ± 0.0116[ab]
	**AL5**	393.17 ± 37.77[ac]	19.73 ± 1.10[a]	**12.67 ± 0.77[c]**	**130.84 ± 19.07[c]**	0.051 ± 0.0104[ab]

Table S7 Numeric data of stomata parameters of Arabidopsis wildtype plants grown *in vitro* with different 2-PG concentrations. Plant were grown in air (400 ppm) on half strength MS media Supplemented with different 2-PG concentrations (0, 10, 50 and 100 µM). After 2-3 weeks stomata parameters were determined by microscopic analysis. Given are means ± SD (Σ 120 stomata per genotype was analyzed, from 4 biological replicates and 30 stomata per leaf) for: **(A)** stomata area, **(B)** stomata length, **(C)** stomata width, **(D)** stomatal density, and **(E)** stomatal index. Values that do not share the same letter (in bold) are significantly different from each other as determined by ANOVA. This data relates to the graphs shown in Fig. 3C of the main manuscript.

**Parameters**	**2-PG content**			
	**0 µM**	**10 µM**	**50 µM**	**100 µM**
**Stomatal area (μm^2)**	676.71 ± 58.28[a]	655.58 ± 43.36[a]	599.72 ± 35.52[ab]	**525.75 ± 18.47[b]**
**Long axis (µm)**	22.93 ± 0.93[a]	22.32 ± 51[ab]	**21.42 ± 0.55[b]**	**20.60 ± 0.95[b]**
**Short axis (µm)**	18.78 ± 0.92[a]	18.70 ± 0.88[a]	17.82 ± 0.64[ab]	**16.26 ± 0.40[b]**
**Stomatal density (mm^2)**	163.68 ± 12.16[a]	150.62 ± 17.88[a]	147.38 ± 14.54[a]	152.14 ± 11.85[a]
**Stomatal index (mm^2 mm^{-2})**	0.111 ± 0.009[a]	0.099 ± 0.012[ab]	**0.088 ± 0.004[bc]**	**0.080 ± 0.006[c]**

Table S8 Numeric data of the metabolite analysis of Arabidopsis lines with GC-specific *PGLP1* overexpression and antisense repression grown in air.

Plants were grown under environmental controlled conditions in air (400 ppm CO_2) or high CO_2 (3000 ppm, for guard cell starch and H_2O_2 control determinations) to growth stage 5.1 (Boyes et al. 2001). Leaf-material was harvested at end of the day (11 h illumination) and analysed by liquid chromatography coupled to tandem mass spectrometry (LC-MS/MS), gas chromatography (GC) and spectrophotometric analysis. Values are means ± SD (n > 5). Values that do not share the same letter (in bold) are significantly different from each other as determined by ANOVA analysis. Selected metabolites (in grey) are displayed in Fig. 4 of the main manuscript. Note, the following total counts are sum parameters including the following metabolites: total amino acids (alanine, arginine, asparagine, cysteine, cystine, glutamate, glutamine, glycine, histidine, isoleucine, leucine, lysine, methionine, phenylalanine, proline, serine, threonine, tryptophan, tyrosine, and valine); total organic acids (aconitate, citrate, fumarate, GABA, isocitrate, lactate, malate, and, succinate); total soluble sugars (glucose, fructose and sucrose)

Metabolites (nmol mg DW^{-1})	Genotype				
	**SL4**	**SL7**	**Col.0**	**AL4**	**A5**
2-PG	**109.6 ± 24.7^{b}**	**102.5 ± 22.1^{b}**	146.6 ± 21.1^{a}	186.9 ± 39.4^{ab}	**223.1 ± 45.9^{b}**
3-PGA	**238.8 ± 37.4^{b}**	**245.4 ± 34.2^{b}**	188.5 ± 52.5^{a}	**155.8 ± 33.4^{c}**	**124.2 ± 30.8^{c}**
AMP	139.0 ± 23.3^{a}	146.6 ± 31.9^{a}	135.9 ± 23.4^{a}	133.3 ± 19.4^{a}	155.1 ± 29.9^{a}
L-Argininosuccinic acid	860.6 ± 253.2^{a}	1099.3 ± 310.1^{a}	692.0 ± 128.7^{a}	916.5 ± 164.3^{a}	924.3 ± 223.3^{a}
NAD^+	**0.9 ± 0.2^{b}**	**0.7 ± 0.2^{b}**	1.2 ± 0.2^{a}	**1.6 ± 0.4^{c}**	**1.8 ± 0.6^{c}**
Ornithine	37.0 ± 8.1^{a}	45.7 ± 10.6^{a}	34.8 ± 11.5^{a}	33.0 ± 4.2^{a}	40.4 ± 7.1^{a}
Alanine	7862.6 ± 2313.2^{a}	9524.2 ± 1409.6^{a}	6597.5 ± 892.4^{a}	7801.2 ± 1118.8^{a}	7376.1 ± 1262.4^{a}
Arginine	4708.1 ± 808.3^{a}	5307.1 ± 1052.2^{a}	3652.5 ± 1356.9^{ab}	**2595.9 ± 661.9^{b}**	**3570.7 ± 516.0^{b}**

Asparagine	9558.1 ±895.5[a]	10823.8 ± 2111.2[a]	8632.0 ± 2529.9[a]	7164.9 ± 559.7[a]	8071.4 ± 1440.4[a]
Citrulline	881.2 ± 219.1[a]	905.5 ± 206.3[a]	772.6 ± 179.7[a]	763.9 ± 101.2[a]	815.4 ± 70.2[a]
Cysteine	27.0 ± 5.8[a]	35.8 ± 8.8[a]	21.9 ± 4.9[a]	33.0 ± 10.1[a]	29.9 ± 9.2[a]
Glutamate	**53169.9 ± 5927.7[b]**	**61024.4 ± 9807.7[b]**	46855.9 ± 2528.5[a]	43559.1 ± 4574.5[a]	44025.3 ± 4471.7[a]
Glutamine	52122.2 ± 5927.7[a]	58725.2 ± 8427.3[a]	42561.3 ± 4082.2[a]	41787.6 ± 4193.4[a]	44441.5 ± 5087.7[a]
Glycine	2006.4 ± 403.9[a]	2063.4 ± 191.8[a]	1901.8 ± 643.6[a]	1924.1 ± 522.5[a]	2119.8 ± 311.2[a]
Histidine	415.6 ± 64.0[a]	432.6 ± 115.4[a]	393.2 ± 59.2[a]	321.3 ± 41.6[a]	331.9 ± 97.5[a]
Isoleucine	**556.7 ± 85.8[b]**	**588.7 ± 99.7[b]**	375.3 ± 40.5[a]	343.7 ± 36.0[a]	398.2 ± 59.1[a]
Leucine	397.6 ± 125.2[ab]	**480.5 ± 81.1[b]**	329.4 ± 72.0[a]	309.6 ± 30.3[a]	377.2 ± 70.5[a]
Lysine	55082.3 ± 5264.5[a]	63290.9 ± 9256.6[a]	47245.0 ± 4532.0[a]	49437.0 ± 5411.1[a]	51043.3 ± 8465.9[a]
Methionine	245.0 ± 68.7[a]	241.4 ± 69.0[a]	190.5 ± 45.5[a]	212.8 ± 28.5[a]	197.39 ± 56.4[a]
Phenylalanine	1545.9 ± 178.3[a]	1642.9 ± 290.6[a]	1342.3 ± 137.4[a]	1195.2 ± 167.5[a]	1227.5 ± 232.2[a]
Proline	12306.2 ± 2680.4[a]	16447.7 ± 7622.0[a]	13302.2 ± 7917.9[a]	11254.5 ± 3115.7[a]	15311.4 ± 6854.3[a]
Serine	15852.8 ± 3319.6[a]	17133.3 ± 4187.7[a]	14418.1 ± 3342.7[a]	13864.9 ± 1737.7[a]	15587.9 ± 2305.6[a]
Threonine	14442.4 ± 2124.4[a]	15778.2 ± 3344.5[a]	11570.8 ± 2613.0[a]	11650.9 ± 1473.3[a]	12728.8 ± 1442.7[a]
Tyrosine	383.9 ± 64.2[b]	400.9 ± 118.53[b]	321.6 ± 51.75[a]	334.6 ± 71.7[a]	331.91 ± 42.7[a]
Valine	2440.2 ± 466.1[a]	2659.56 ± 479.1[a]	2187.3 ± 265.3[a]	2255.6 ± 319.8[a]	2246.1 ± 296.2[a]
Succinate	185.8 ± 54.3[a]	202.9 ± 54.8[a]	221.1 ± 94.8[a]	202.9 ± 75.4[a]	222.26 ± 87.6[a]
Cis-Aconitate	74.9 ± 23.3[a]	85.1 ± 17.8[a]	72.3 ± 13.0[a]	74.8 ± 11.9[a]	80.1 ± 17.5[a]
Citrate	55237.1 ± 18497.9[a]	68562.4 ± 16030.2[a]	60521.5 ± 11455.6[a]	66322.2 ± 18514.8[a]	71861.4 ± 15682.7[a]
Fumarate	1297.7 ± 444.3[a]	1371.2 ± 408.1[a]	1375.3 ± 427.7[a]	1396.1 ± 671.1[a]	1369.9 ± 604.8[a]
GABA	860.6 ± 253.2[a]	1099.3 ± 310.1[a]	691.9 ± 128.68[a]	916.5 ± 164.3[a]	924.3 ± 223.3[a]
Isocitrate	**149510.8 ± 22324.8[b]**	**149486.5 ± 28788.3[b]**	112278.6 ± 11777.34[a]	94837.6 ± 9928.2[a]	99599.0 ± 13170.0[a]
Lactate	194.5 ± 48.7[a]	198.3 ± 51.0[a]	168.6 ± 32.6[a]	149.8 ± 17.2[a]	130.6 ± 29.1[a]
Malate	172994.0 ± 21582.5[a]	178744.9 ± 33678.9[a]	169377.1 ± 24954.2[a]	153166.0 ± 39250.2[a]	157756.8 ± 40986.4[a]

**Total contents (nmol mg DW^{-1})**					
Total amino acids	**158.98 ± 16.36^{b}**	**155.36 ± 13.41^{b}**	133.15 ± 14.50^{a}	**117.19 ± 13.32^{c}**	127.22 ± 10.34^{a}
Total organic acids	**319.13 ± 26.99^{b}**	**324.49 ± 49.29^{b}**	253.85 ± 41.03^{a}	210.03 ± 49.65^{a}	**203.06 ± 38.17^{b}**
**Soluble sugars (µg g DW^{-1})**					
Glucose	**2.48 ± 0.63^{b}**	**2.52 ± 0.53^{b}**	1.61 ± 0.20^{a}	**1.07 ± 0.18^{c}**	**1.15 ± 0.19^{c}**
Fructose	**1.27 ± 0.10^{b}**	**1.22 ± 0.14^{b}**	0.92 ± 0.12^{a}	**0.69 ± 0.11^{c}**	**0.64 ± 0.16^{c}**
Sucrose	**4.41 ± 0.27^{b}**	**4.47 ± 0.49^{b}**	3.78 ± 0.28^{a}	3.72 ± 0.33^{a}	3.82 ± 0.30^{a}
Total soluble sugars	**8.44 ± 0.78^{b}**	**8.09 ± 0.87^{b}**	6.30 ± 0.39^{a}	**5.26 ± 0.71^{c}**	**5.32 ± 0.54^{c}**
Starch (µmol g DW^{-1})	75.75 ± 3.99^{a}	80.22 ± 21.85^{a}	85.12 ± 28.16^{a}	85.51 ± 20.66^{a}	86.08 ± 25.33^{a}
**Guard cell specific metabolites (area per stomata µm^{-2}/ signal area per unit stomata µm^{-2} µm^{-2})**					
Starch (air)	**20.52 ± 1.69^{b}**	**21.61 ± 1.56^{b}**	17.26 ± 1.13^{a}	**12.16 ± 2.78^{c}**	**11.86 ± 1.05^{c}**
Starch (high CO_2)	30.83 ± 4.08^{d}	32.15 ± 1.93^{d}	31.11 ± 4.10^{d}	29.63 ± 5.82^{d}	28.70 ± 2.62^{d}
H_2O_2 (air)	**0.0239 ± 0.0019^{b}**	**0.0224 ± 0.0028^{b}**	0.0349 ± 0.0027^{a}	**0.0445 ± 0.0037^{c}**	**0.0449 ± 0.0034^{c}**
H_2O_2 (high CO_2)	0.061 ± 0.007^{d}	0.062 ± 0.004^{d}	0.061 ± 0.010^{d}	0.062 ± 0.004^{d}	0.049 ± 0.007^{d}

Table S9. Primers used for PCR amplification.

Underlined sequences indicate the introduced restriction sites in the primers used to produce expression constructs. ATG in bold print highlight the start codon for methionine.

Stock Number	Name	Sequence (5´-to3´)
P967	*AtSBPase*_fw_XmaI	CCCGGG**ATG**GAGACCATCGCGTGC
P968	*AtSBPase*_rev_XmaI	CCCGGGCTAAGCGGTAACTCCAATGGG
P953	*SlPGLP1*_fw	ATGCTAAGCATCAGAGTAACAGCAACC
P954	*SlPGLP1*_rev	AACTGCAGCCGCTTTAATGGAGAG
P444	*S16*-fw	GGCGACACAACCAGCTACTGA
P445	*S16*-rev	CGGTAACTCTTCTGGTAACGA
P950	*AtGC1*_S1141_SacI	GAGCTC**ATG**GTTGCAACAGAGAGGATGAAT
P951	*AtGC1*-AS-BamHI	GGATCCATTTCTTGAGTAGTGATTTTGAAG
P974	*SlPGLP*-fw-Short	GGTTCAATGGTTGGTGCGATCC
P975	*SlPGLP*-rev-End	GGATCCTCATTTTTCGAACTGC
P977	*AtPGLP*-fw-Short	GGCTCTATGGTTGGTGCTCTTG
P978	*AtPGLP*-rev-End	CGGAGACAGAAAATCGGAGATC
